# Multidimensional characterization of heterogeneity in pesticide co-exposure patterns

**DOI:** 10.3389/fchem.2026.1890266

**Published:** 2026-08-14

**Authors:** Han Su, Lezhen Zhang, Wenjun Tian, Bingru Lu, Xiangrui Guo, Yiqing Liu

**Affiliations:** 1 Department of Clinical Laboratory, Shandong Provincial Hospital Affiliated to Shandong First Medical University, Jinan, Shandong, China; 2 Jinan Dian Medical Laboratory Co., Ltd., Jinan, Shandong, China

**Keywords:** agricultural population, co-occurrence network, mass spectrometry analysis, multidimensional heterogeneity, pesticide co-exposure

## Abstract

**Objectives:**

This study analyzed clinical data from 1641 pesticide-exposed patients screened for a total of 159 pesticide compounds admitted to Shandong First Medical University Affiliated Provincial Hospital from 1 January 2023 to 31 December 2024, aiming to elucidate the multidimensional heterogeneity of pesticide co-exposure patterns.

**Methods:**

Clinical information and biological samples were collected from all participants. Pesticide detection was performed using Agilent 7890B-7250 Q-TOF/MS and AB SCIEX Triple Quad 4500MD mass spectrometry systems. Seasonal, gender-specific, and age-group variations in pesticide exposure were assessed, and a co-occurrence network was constructed to evaluate concurrent exposure patterns and concentration distributions.

**Results:**

Herbicides exhibited the highest exposure prevalence within their respective tested sub-cohorts. Specifically, glyphosate showed a targeted detection frequency of 49.32% (144/292) among the screened individuals. Concentration peaks for glyphosate and paraquat occurred between July and September, with median levels reaching 3.42 and 3.15 log_10_ μg/L, respectively. Pesticide exposure displayed a bimodal age distribution, with a secondary peak among individuals aged 15–20 years and a primary peak in those aged 55–60 years. In the 40–55 age group, male exposure prevalence was significantly higher than female (male-to-female ratio: 2.8:1). Network analysis identified 38 significant co-occurrence patterns (r > 0.5, P < 0.0016), with the most prominent involving glyphosate, imidacloprid, and metolachlor. Log-transformed pesticide concentrations revealed marked differences in variability across chemical classes. Herbicides (e.g., glyphosate, paraquat) and certain broad-spectrum insecticides (e.g., chlorfenapyr) showed highly right-skewed concentration distributions, whereas tebuconazole and cinosulfuron exhibited relatively concentrated profiles. Thiamethoxam demonstrated substantially greater concentration variability compared to other compounds, indicating pronounced heterogeneity in its application patterns. Collectively, these findings highlight complex heterogeneity in pesticide exposure across temporal, demographic, and chemical dimensions.

**Conclusion:**

This study reveals distinct multidimensional heterogeneity in pesticide exposure, providing critical evidence for developing targeted intervention strategies. Tailored protective measures based on seasonal trends, gender-age subgroups, and prevalent co-exposure patterns may more effectively mitigate pesticide-related risks among agricultural populations.

## 1 Introduction

As a major agricultural province in China, Shandong consumed 105,000 tons of pesticides in 2022, accounting for 42% of the national total (Xiangguo, 2024). The routine combined use of herbicides and insecticides in cultivating staple crops such as wheat, corn, and vegetables has led to regionally distinctive human exposure profiles (Wu and Chen, 2025). However, this extensive usage poses significant environmental and public health challenges that have drawn international concern. Pesticide residues are ubiquitously present in soil, water, and air, and can enter the human body through bioaccumulation along the food chain. Both chronic low-dose and acute high-level exposures have been consistently linked to various adverse health outcomes, including endocrine disruption, neurotoxicity, and increased risks of certain cancers (Bao et al., 2020; Supanekar, 2022; Beyuo et al., 2024). Therefore, comprehensive investigation into the distribution characteristics of pesticide exposure and associated health implications is essential for guiding science-based pesticide management policies, targeted risk communication to vulnerable groups, and effective environmental protection initiatives. Since the launch of China’s “Zero Growth Action Plan for Fertilizer and Pesticide Use by 2020” in 2015, Shandong has witnessed substantial shifts in pesticide utilization patterns. Beyond the regulatory shift toward lower-toxicity synthetic agents, nature-derived biopesticides are emerging as a promising Frontier in sustainable pest management. Suo et al. (2026) demonstrated that a farnesal nanoemulsion biopesticide achieves high efficacy against aphids while exhibiting remarkable environmental safety, offering a novel paradigm for pesticide utilization strategies (Suo et al., 2026). Specifically, the use of highly toxic organophosphorus pesticides has declined by 38.6%, while applications of lower-toxicity, broad-spectrum agents such as glyphosate have risen by 52.3%.The World Health Organization (WHO) has long pursued two key objectives related to pesticides: the prohibition of the most toxic compounds to humans and those with the greatest environmental persistence, and the establishment of maximum residue limits for pesticides in food and water to safeguard public health. These regulatory shifts have significantly influenced population-level exposure patterns. Nevertheless, substantial challenges remain in identifying clear or potential pesticide exposures globally due to analytical complexities of multi-residue detection, lack of standardized regulatory thresholds for co-exposures, and fragmented surveillance systems in clinical settings (WHO, 2025). On one hand, the complex distribution of pesticide residues in environmental and biological matrices complicates detection, increasing both technical difficulty and operational costs (López-Maldonado et al., 2025). On the other hand, due to their inherent toxicity and widespread availability, particularly in low- and middle-income countries, pesticides are frequently used in intentional self-poisoning incidents, representing a leading cause of suicide by poisoning (Jallow et al., 2017). This dual role exacerbates disparities in the regulation, production, sale, and use of pesticides.

In recent years, mass spectrometry techniques—particularly liquid chromatography-tandem mass spectrometry (LC-MS/MS) and gas chromatography-mass spectrometry (GC-MS)—have become widely adopted in pesticide detection due to their high sensitivity, selectivity, and throughput (López-Maldonado et al., 2025). While mass spectrometry remains the gold standard for pesticide residue analysis, alternative detection platforms such as complex-based probes utilizing coordination water and supramolecular chains have recently emerged as promising complementary tools for specific pesticide recognition (Li et al., 2026). Additionally, emerging nanomaterial-based sensing approaches, including theoretical investigations of 2D materials for organochlorine pesticide detection, represent alternative strategies for environmental monitoring (Qin et al., 2023). Their ability to simultaneously identify and quantify multiple pesticide residues greatly enhances detection efficiency and provides robust quantitative data essential for risk assessment. As a result, many national and regional food safety authorities have incorporated these methods into standardized pesticide residue testing protocols. For example, the European Union’s SANTE guidelines explicitly validate the applicability of LC-MS/MS for pesticide residue analysis (Commission, 2024).

This study aims to systematically analyze clinical data on pesticide detections across different quarters using mass spectrometry techniques, to reveal seasonal variation patterns in pesticide exposure among susceptible populations, assess potential health implications, provide more accurate data support for pesticide residue risk assessment, and inform evidence-based public health policy development. Crucially, while prior research has significantly advanced our understanding of specific compounds, many studies have largely focused on single-pesticide analyses or isolated chemical classes (Bao et al., 2020), which may fail to capture the synergistic toxicities inherent in real-world ‘pesticide cocktails' (Antonangeli et al., 2023; Wolfe and Marsit, 2023). Unlike prior studies that focused on community-based biomonitoring in specific occupational groups (Barrón Cuenca et al., 2020), this study utilizes a large-scale clinical dataset to map multidimensional co-exposure networks across a diverse patient population, filling a gap in hospital-based acute and sub-acute exposure surveillance (Bao et al., 2020).

## 2 Materials and methods

### 2.1 General information

This study enrolled a total of 1,641 patients who underwent pesticide testing at Shandong Provincial Hospital Affiliated to Shandong First Medical University between 1 January 2023, and 31 December 2024, including samples from both outpatient and inpatient settings. Inclusion criteria were as follows: (1) patients undergoing their first toxicological screening; (2) patients with a documented history of toxic exposure; or (3) patients without a clear exposure history, adjudicated via multidisciplinary team (MDT) consultation based on objective toxidromes (e.g., miosis, cholinergic signs) or depressed acetylcholinesterase activity. Exclusion criteria included: (1) patients who had undergone retesting following an initial toxicological assessment; (2) patients with known exposure to pharmaceuticals requiring therapeutic drug monitoring; (3) asymptomatic individuals requesting routine toxicological testing; and (4) patients with incomplete clinical records. The final cohort was selected according to these predefined inclusion and exclusion criteria.

This study has been approved by Biomedical Research Ethic Committee of Shandong Provincial Hospital Affiliated to Shandong First Medical University (No. 2026-030). The requirement for informed consent was waived by the Biomedical Research Ethic Committee of Shandong Provincial Hospital Affiliated to Shandong First Medical University because of the retrospective nature of the study.

### 2.2 Instruments and reagents

#### 2.2.1 Instruments

The analytical instruments used included an Agilent 7890B-7250 Q-TOF/MS gas chromatography-mass spectrometry (GC-MS) system, an AB SCIEX Triple Quad 4500MD liquid chromatography-tandem mass spectrometry (LC-MS/MS) analyzer, a Kinetex® 5 μm EVO C18 100 Å LC column (100 × 2.1 mm), a Carbon/NH2 solid-phase extraction column (Agilent, USA), a Milli-Q ultrapure water system (Millipore, USA), an MD200-2 nitrogen evaporator (Aosheng, China), a Thermo high-speed refrigerated centrifuge, a vortex mixer (Vortex, USA), a PL602-L electronic balance (Mettler-Toledo, Switzerland), Eppendorf pipettes (USA), and 1.5 mL and 2.0 mL microcentrifuge tubes (Axygen, USA).

#### 2.2.2 Reagents

Pesticide standards, quality control materials, and calibration solutions were purchased from Qingdao Huikang Bioengineering Co., Ltd. Methanol, ethyl acetate, and acetonitrile (HPLC grade) were obtained from Merck (Germany).

#### 2.2.3 Method validation

For the GC-MS/MS analysis of pyrethroid pesticides, heptachlor epoxide B was employed as the internal standard. For the LC-MS/MS analysis of the remaining 151 pesticides, although isotopically labeled internal standards were not utilized for all target analytes, the accuracy and precision of the results were ensured through matrix-matched calibration using whole blood or plasma, supplemented by the performance validation parameters detailed below. The spiked recoveries for all detected pesticides fell within the acceptable range of 85%–115%, satisfying the accuracy requirements for quantitative analysis. The intra-day and inter-day precision of the method was evaluated using the coefficient of variation (CV). The results demonstrated that the intra-day and inter-day CVs were ≤ 20% at concentrations near the lower limit of quantification (LLOQ), and ≤ 15% at all other concentration levels, indicating good repeatability and stability of the method. The linear regression correlation coefficients (r) for all target pesticides were ≥ 0.99, demonstrating excellent linearity across a broad concentration range.

### 2.3 Sample collection and preparation

Peripheral venous blood (5 mL) was collected from each participant upon admission. For the quantification of 159 target compounds, a stratified matrix approach was implemented: plasma (separated via centrifugation at 3,500 rpm, 4 °C for 10 min) was utilized for pyrethroids, paraquat, diquat, glyphosate, and glufosinate, while whole blood was used for the remaining 147 pesticides to ensure optimal analytical sensitivity. All specimens were stored at −80 °C prior to LC-MS/MS or GC-MS/MS analysis.

### 2.4 Detection method

GC-MS/MS parameters:8 pesticide residues (Cyfluthrin, Cypermethrin, Fenpropathrin, Fenvalerate, Flucythrinate, Lambda-cyhalothrin, permethrin, Esfenvalerate) were analyzed with an Agilent 7890B-7250 Q-TOF/MS gas chromatography-mass spectrometry (GC-MS) system(Agilent, Santa Clara, CA). The chromatographic separation of these 8 pesticide residues were performed on a HP-5ms column (30 m by 0.25 mm by 0.25 μm; Agilent). The oven was kept at an initial temperature of 60 °C for 1 min; heated to 170 °C at a rate of 40 °C/min, and finally to 310 °C at a rate of 10 °C/min; and then maintained at this temperature for 8 min. The inlet and transfer line temperatures were 270 °C and 310 °C, respectively. Helium was used as the carrier gas for GC at a flow rate of 0.95 mL/min. The injection volume was 1 μL, and the solvent delay was 2.5 min for the mass selective detector. The ion source and quadruple temperatures were 250 °C and 150 °C, respectively. The MS/MS parameters were optimized by a full scan (Supplementary Table S2). The mass range (m/z) was 50–550 amu for the full scan.

LC-MS/MS parameters: Glufosinate and Glyphosate were chromatographically separated on an Anionic Polar Pesticide column (100 × 2.1 mm, 5 μm; Waters). The mobile phase was composed of solvent A (0.9% formic acid in water) and solvent B (0.9% formic acid in acetonitrile), delivered according to the following gradient program: 0.0–3.0 min, linear increase from 40% A to 85% A; 3.0–6.0 min, maintained at 85% A; 6.0–6.5 min, rapid decrease to 40% A (B-curve: 6); and 6.5–8.0 min, held at 40% A for system re-equilibration. The flow rate was 0.6 mL/min, the injection volume was 10 μL, and the column temperature was maintained at 50 °C. Diquat and Paraquat were chromatographically separated on a HILIC column (100 × 2.1 mm, 2.6 µm; Kinetex). The mobile phase consisted of solvent A (1 mmol/L ammonium acetate and 0.4% formic acid in water) and solvent B (acetonitrile), with the following gradient elution program: 0.0–0.5 min, maintained at 20% A; 0.5-6.0 min, linear increase to 90% A; 6.0-10.0 min, held at 90% A; 10.0–10.1 min, returned to initial 20% A; followed by a 1.9 min re-equilibration period (10.1–12.0 min). The flow rate was 0.3 mL/min, the injection volume was 10 μL, and the column temperature was maintained at 40 °C. The remaining 147 pesticides were chromatographically separated on an EVO C18 column (100 × 2.1 mm, 5 μm; Kinetex). The mobile phase comprised solvent A (5 mmol/L ammonium acetate in water) and solvent B (5 mmol/L ammonium acetate in methanol), with gradient elution performed as follows: 0.0–1.0 min, 98% A; 1.0-10.0 min, linear decrease to 0% A; 10.0-13.0 min, held at 0% A; 13.0–13.1 min, rapid increase to 98% A; and 13.1–15.0 min, 98% A for equilibration. The flow rate was 0.6 mL/min, the injection volume was 5 μL, and the column temperature was maintained at 40 °C. All the parameters shown as Supplementary Tables S1, S2.

### 2.5 Observation indicators

Clinical data, including pesticide detection results and patient age, were retrospectively extracted from the hospital laboratory’s electronic medical record system for patients meeting the inclusion criteria. A sample was classified as positive if target pesticide compounds were detected and quantifiable in the biological specimen; otherwise, it was recorded as negative. The specific identities and names of all detected and non-detected pesticides were systematically documented.

### 2.6 Statistical analysis

Data were analyzed using SPSS 30.0 (IBM, USA) and GraphPad Prism 9.5 (GraphPad Software, USA). Continuous variables exhibiting non-normal distribution, assessed by the Shapiro-Wilk test (p < 0.05)—including pesticide concentrations—were summarized as median and interquartile range (IQR). Group comparisons were performed using the Kruskal–Wallis test with post hoc multiple comparison adjustments. Categorical variables were presented as frequencies and proportions. Co-exposure networks were constructed based on Pearson correlation coefficients (r), retaining only pairs with |r| ≥ 0.5 and p < 0.01 after false discovery rate (FDR) correction. To balance stringency and sensitivity across the 159-pesticide dataset, a dual-correction framework was implemented: (1) univariate prevalence comparisons (Table 1) utilized a Bonferroni-corrected threshold p < 0.0016) to minimize Type I errors; (2) co-exposure networks were constructed in GraphPad Prism using Pearson correlation coefficients (|r|≥0.5) with FDR controlled at p < 0.01 via the Benjamini–Hochberg procedure. All statistical tests were two-sided (α = 0.05).

**TABLE 1:** Clinical data of pesticide exposure in the study population.

Variable	N	Negative N (%)	Positive				
			Q1 (Median, IQR)	Q2 (Median, IQR)	Q3 (Median, IQR)	Q4 (Median, IQR)	P value
Age	1641	552 (33.63%)	198 (52, 31∼63)	353 (53, 34∼65)	322 (51, 32∼65)	216 (50.5, 31∼61.75)	0.833
Gender	1641	552 (33.63%)	198	353	322	216	-
Male	846	274 (32.38%)	95	177	187	113	<0.001
Female	795	278 (34.96%)	103	176	135	103	<0.001
Acetamiprid	126	105 (83.33%)	5 (23.73, 3.51∼5981)	7 (19.69, 2.07∼1754)	6 (3.28, 1.84∼309.7)	3 (63.99, 12.3∼2780)	0.990
Acetochlor	96	48 (50%)	14 (18.25, 2.201∼68.81)	20 (191, 17.59∼682)	9 (25.85, 15.02∼77.43)	5 (14.8, 9.63∼147.5)	0.072
Alachlor*	49	48 (97.95%)	​	1 (12.75, 12.75∼12.75)	​	​	0.730
Ametryn	52	48 (92.30%)	​	1 (20.46, 20.46∼20.46)	1 (363.8, 363.8∼363.8)	2 (1692, 11.07∼3374)	0.205
Amitraz*	189	188 (99.47%)	​	1 (11.18, 11.18∼11.18)	​	​	0.539
Atrazine	140	105 (75%)	5 (54.47, 27.93∼104.8)	8 (18.73, 0.6425∼454.9)	17 (13.75, 3.76∼115.7)	5 (100.9, 1.677∼414.1)	0.114
Avermectin	65	10 (15.38%)	4 (28.84, 18.39∼299.7)	20 (27.23, 19.17∼75.55)	20 (11.3, 2 .692∼61.04)	11 (32.48, 20.75∼135.4)	<0.001
Azoxystrobin*	51	48 (94.11%)	1 (397.8, 397.8∼397.8)	1 (26.18, 26.18∼26.18)	1 (4.17, 4.17∼4.17)	​	0.796
Bifenthrin	242	226 (93.38%)	4 (142.6, 25.46∼686.1)	4 (71.31, 18.03∼334.5)	5 (56.01, 15.49∼138.9)	3 (31.36, 17.08∼71.27)	0.889
Buprofezin*	106	105 (99.05%)	​	1 (52.16, 52.16∼52.16)	​	​	0.526
Butralin	110	105 (95.45%)	2 (475.6, 241.7∼709.5)	1 (1.82, 1.82∼1.82)	2 (334.4, 126.4∼542.4)	​	0.628
Carbendazim*	4	2 (50%)	​	​	2 (0.83, 0.38∼1.28)	​	0.083
Carbofuran	117	105 (89.74%)	4 (111.4, 17.19∼461.9)	6 (187.5, 13.47∼1356)	1 (0.56, 0.56∼0.56)	1 (3.74, 3.74∼3.74)	0.213
Chlorfenapyr	42	3 (7.142%)	5 (8.16, 1.13∼68.89)	12 (109.7, 35.01∼268)	14 (181, 28.16∼495.7)	8 (34.02, 5.608∼135.8)	0.001
Chlorpyrifos	209	188 (89.95%)	5 (69.8, 28.55∼355)	8 (79.75, 14.14∼458.5)	6 (246.4, 21.21∼387)	2 (148.1, 16.65∼279.5)	0.763
Cinosulfuron*	106	105 (99.05%)	1 (2, 2∼2)	​	​	​	0.335
Cyanazine	52	48 (92.30%)	​	1 (188, 188∼188)	1 (2111, 2111∼2111)	2 (1292, 10.06∼2575)	0.205
Cyfluthrin*	2	0 (0%)	​	​	1 (4.51, 4.51∼4.51)	1 (2.47, 2.47∼2.47)	1
Cypermethrin	59	41 (69.49%)	1 (0.89, 0.89∼0.89)	3 (51, 34.21∼358.3)	7 (101.8, 47.84∼290.6)	7 (36.39, 28.68∼229.3)	0.186
Deltamethrin*	189	188 (99.47%)	​	​	1 (11.25, 11.25∼11.25)	​	0.501
Dichlorvos	223	105 (47.08%)	25 (71.76,23.22∼235.9)	30 (42.26, 10.55∼110.6)	43 (26.33, 14.8∼172.7)	20 (10.77, 6.348∼38.71)	0.214
Difenoconazole	53	48 (90.56%)	1 (7.57, 7.57∼7.57)	1 (10.47, 10.47∼10.47)	3 (2.54, 1.01∼125.1)	​	0.202
Dimethoate	193	188 (97.40%)	1 (2.57, 2.57∼2.57)	2 (42097, 94.55∼84100)	​	2 (36250, 0.84∼72500)	0.365
Diquat	439	245 (55.80%)	32 (526.5, 53.47∼2011)	57 (1242, 85.66∼7340)	62 (494.2, 85.26∼5146)	43 (545.6, 133∼4032)	0.006
Diuron*	107	105 (98.13%)	​	​	​	2 (67.3, 41.01∼93.58)	0.010
Esfenvalerate*	42	41 (97.61%)	​	1 (185.3, 185.3∼185.3)	​	​	0.450
Fenobucarb*	49	48 (97.95%)	​	​	1 (49.81, 49.81∼49.81)	​	0.354
Fenpropathrin*	44	41 (93.18%)	2 (373.8, 0.24∼747.3)	​	​	1 (5.13, 5.13∼5.13)	0.021
Fenvalerate*	43	41 (95.34%)	​	2 (462.6, 318.5∼606.8)	​	​	0.145
Fipronil*	106	105 (99.05%)	​	​	1 (122, 122∼122)	​	0.533
Fludioxonil*	50	48 (96%)	​	​	1 (73.7, 73.7∼73.7)	1 (2.64, 2.64∼2.64)	0.308
Glufosinate	224	148 (66.07%)	14 (402, 76.11∼4519)	30 (991, 153.8∼3672)	16 (1324, 65.81∼12670)	16 (78.86, 18.26∼1567)	<0.001
Glyphosate	292	148 (50.68%)	21 (734.5, 68.26∼18520)	44 (988.1, 90.4∼30140)	41 (3725, 268.4∼12750)	38 (4005, 166.3∼21745)	<0.001
Hexazinone*	107	105 (98.13%)	1 (62880, 62880∼62880)	1 (22176, 22176∼22176)	​	​	0.611
Imidacloprid	135	105 (77.77%)	6 (1474, 27.62∼7716)	16 (2100, 12.9∼10323)	6 (226.1, 37.11∼9352)	2 (1387, 335∼2439)	0.037
Isofenphos-methy*	6	3 (50%)	1 (2.4, 2.4∼2.4)	2 (106.4, 46.23∼166.5)	​	​	0.006
Isoprocarb*	49	48 (97.95%)	​	1 (1580, 1580∼1580)	​	​	0.730
Lambda-cyhalothrin	79	41 (51.89%)	3 (22.29, 18.92∼80.51)	13 (285.3, 103.4∼650.6)	9 (22.04, 6.221∼39.78)	13 (88.2, 19.34∼215.8)	0.979
Malathion*	5	3 (60%)	​	1 (41.58, 41.58∼41.58)	1 (1874, 1874∼1874)	​	0.011
Metalaxyl*	107	105 (98.13%)	1 (10150, 10150∼10150)	​	1 (1.06, 1.06∼1.06)	​	0.620
Methomyl	118	105 (88.98%)	1 (2705, 2705∼2705)	7 (182.7, 95.3∼559.9)	2 (106.5, 15.97∼197)	3 (189.8, 0.55∼314.7)	0.147
Metolachlor	56	48 (85.71%)	​	5 (109.7, 12.78∼3387)	2 (385.8, 24.64∼746.9)	1 (1.02, 1.02∼1.02)	0.497
Omethoate	23	3 (13.04%)	4 (6959, 6.44∼44862)	6 (30144, 19.3∼96740)	6 (92.71, 6.213∼31477)	4 (35990, 125.9∼102610)	0.002
Paraquat	373	245 (65.68%)	25 (4527, 359.8∼8686)	38 (1705, 298∼8138)	38 (7093, 1073∼25654)	27 (2696, 349.5∼8715)	0.013
Pendimethalin	56	48 (85.71%)	4 (16.77, 5.33∼165.8)	3 (7.47, 5.2∼115.1)	1 (24.52, 24.52∼24.52)	​	0.102
Permethrin*	42	41 (97.61%)	​	​	​	1 (1.24, 1.24∼1.24)	0.724
Phorate	30	3 (10%)	10 (28.6, 9.225∼665.6)	5 (289.2, 7.54∼2160)	6 (93.85, 5.836∼1565)	6 (63.89, 27.5∼177.7)	0.011
Phorate sulfone	11	3 (27.27%)	1 (113.6, 113.6∼113.6)	​	2 (83.66, 69.55∼97.76)	5 (94.57, 21.67∼182.8)	0.399
Phorat-sulfoxide	12	3 (25%)	1 (582.5, 582.5∼582.5)	​	2 (292.8, 100.3∼485.2)	6 (187, 21.14∼999.3)	0.542
Phoxim	217	188 (86.63%)	6 (574.9, 166.8∼1314)	12 (48.05, 3.428∼126.4)	9 (28.37, 2.985∼496.4)	2 (5.15, 3.58∼6.72)	0.377
Piperonyl butoxide*	189	188 (99.47%)	​	​	1 (28.2, 28.2∼28.2)	​	0.508
Prochloraz	107	105 (98.13%)	​	1 (590.9, 590.9∼590.9)	1 (4.842, 4.842∼4.842)	​	0.752
Prometryn*	107	105 (98.13%)	​	​	1 (2.33, 2.33∼2.33)	1 (12.97, 12.97∼12.97)	0.408
Propazine	53	48 (90.56%)	1 (6.33, 6.33∼6.33)	​	2 (17.04, 2.65∼31.43)	2 (8.04, 2.03∼14.05)	0.179
Pymetrozine	109	105 (96.33%)	​	​	3 (30280, 29.86∼32380)	1 (2922, 2922∼2922)	0.178
Pyraclostrobin	52	48 (92.30%)	​	1 (0.11, 0.11∼0.11)	3 (61.64,9.13∼328.8)	​	0.083
Pyridaben	52	48 (92.30%)	​	1 (126.4, 126.4∼126.4)	1 (107.7, 107.7∼107.7)	2 (126.8, 79.29∼174.3)	0.208
Quinalphos*	189	188 (99.47%)	1 (3.85, 3.85∼3.85)	​	​	​	0.298
Rotenone*	49	48 (97.95%)	​	​	​	1 (10.62, 10.62∼10.62)	0.125
Simazine*	107	105 (98.13%)	​	​	​	2 (2.241, 2.2∼2.282)	0.010
Simetryn*	49	48 (97.95%)	​	​	​	1 (7.4, 7.4∼7.4)	0.125
Sulfotep*	189	188 (99.47%)	​	1 (1.27,1.27∼1.27)	​	​	0.539
Tebuconazole	195	188 (96.41%)	​	3 (65.4, 2.8∼196)	3 (2.18, 2.04∼11.04)	1 (1875, 1875∼1875)	0.521
Thiamethoxam	29	2 (6.896%)	2 (4998, 0.311∼9995)	15 (265.1, 1.362∼9780)	8 (6.015, 2.248∼1539)	2 (103.4, 1.51∼205.2)	0.004
Thiophanate-methyl*	51	48 (94.11%)	​	3 (4.72, 0.52∼16.51)	​	​	0.265
Tralopril	39	3 (7.692%)	9 (257.7, 2.48∼2492)	10 (177.5, 7.885∼408.2)	9 (174.9, 23.16∼1312)	8 (271.6, 14.25∼1498)	0.007
Triadimenol*	108	105 (97.22%)	​	3 (82.29, 6.89∼257.5)	​	​	0.080
Triazophos*	190	188 (98.94%)	1 (6.17, 6.17∼6.17)	1 (45.19, 45.19∼45.19)	​	​	0.587
Trichlorfon	150	5 (3.333%)	27 (248.4, 86.38∼545.5)	41 (108, 6.813∼524.1)	52 (77.96, 23.14∼272.4)	25 (56.01, 6.175∼244)	0.004
Triforine*	190	188 (98.94%)	​	​	1 (11.14, 11.14∼11.14)	1 (7.2, 7.2∼7.2)	0.457

N: Total number tested per pesticide; Q1-Q4: Calendar quarters (Q1: Jan-Mar; Q2: Apr-Jun; Q3: Jul-Sep; Q4: Oct-Dec); Median (IQR): Median concentration and interquartile range (μg/L); P-value: Kruskal–Wallis test with Bonferroni correction for multiple comparisons (significance threshold: P < 0.0016, 0.05/32 tests). LOD: Limit of detection defined as signal-to-noise ratio (S/N) of 3:1; LOQ: Limit of quantification defined as S/N of 10:1; ND: Not detected (S/N < 3). Missing values were excluded from statistical analyses. The following pesticides were not detected in any of the samples: Etofenprox, Flucythrinate, Linuron, Metazachlor, Metribuzin, Pyrazosulfuron-ethyl, Bensulfuron-methyl, Benzoximate, Fenoxaprop-P-ethyl, Fenpropidin, Fenamidone, Ethiprole, Epoxiconazole, Diflubenzuron, Fenarimol, Cyclosulfamuron, Chlortoluron, Chlorsulfuron, Carbaryl, Bifenox, Cymoxanil, Flufenoxuron, Pretilachlor, Phenthoate, Oxamyl, Oxadiargyl, Molinate, Flutriafol, Flutolanil, Imidaclothiz, Iprobenfos, Isoproturon, Ethirimol, Dodine, Cyprodinil, Carboxin, Flusilazole, Phosfolan, Phosmet, Phosalone, Phosphamidon, Propargite, Profenofos, Pirimiphos-methyl, Parathion, Monocrotophos, Methidathion, Isazofos, Fenthion, Imazaquin, Fenamiphos, Aldicarb, Azinphos-methyl, Coumaphos, Chlorpyrifos-methyl, Diazinon, Dicrotophos, Edifenphos, Ethion, Clofentezine, Ethoprophos, Propanil, Propachlor, Pirimicarb, Penconazole, Paclobutrazol, Propyzamide, Oxadiazon, Mefenacet, Ivermectin B1a, imazalil, Hexythiazox, Hexaflumuron, Napropamide, Pyriftalid, Thiobencarb, Thidiazuron, Triadimefon, Thiabendazole, Terbuthylazine, Teflubenzuron, Spiroxamine (isomer), Spirodiclofen, Pyrimethanil, Hexaconazole, Bitertanol, Uniconazole, Triasulfuron, Benalaxyl. *Due to the extremely small number of positive cases (n = 1–3), the median and IQR for these pesticides are for reference only and lack population representativeness.

## 3 Results

### 3.1 Representative chromatograms for multi-residue pesticide screening

**FIGURE 1:**
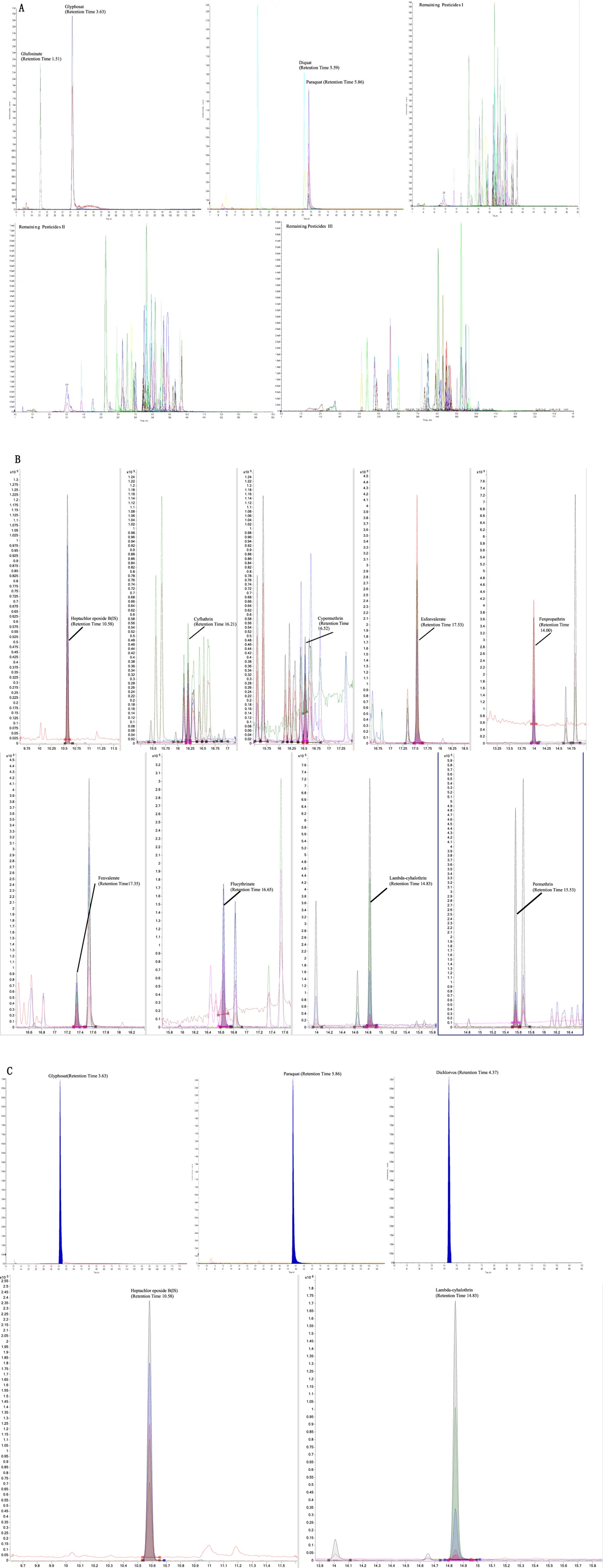
Representative chromatograms for multi-residue pesticide screening. **(A)** Mix Standard of LC-MS Chromatograms: Total ion chromatogram (TIC) showing the well-resolved peaks of targeted pesticides involved Glufosinate and Glyphosat, Diquat and Paraquat. Remaining Pesticides I(Amitraz, Azinphos-methyl, Chlorpyrifos, Chlorpyrifos-methyl, Clofentezine, Coumaphos, Deltamethrin, Diazinon, Dicrotophos, Dimethoate, Epoxiconazole, Ethiprole, Etofenprox, Fenamiphos, Fenarimol, Fenthion, Isazofos, Isofenphos-methy, Malathion, Methidathion, Monocrotophos, Omethoate, Phenthoate, Phorate, Phorate Sulfone, Phorate sulfoxide, Phosalone, Phosfolan, Phosmet, Phosphamidon, Phoxim, Pirimicarb, Profenofos, Propargite, Quinalphos, Sulfotep, Tebuconazole, Triazophos, Triforine, Avermectin, Chlorfenapyr, Tralopril). Remaining Pesticides II(Acetamiprid, Atrazine, Bensulfuron-methyl, Benzoximate, Bifenox, Bifenthrin, Buprofezin, Butralin, Carbaryl, Carbofuran, Chlorsulfuron, Chlortoluron, Cinosulfuron, Cyclosulfamuron, Cymoxanil, Dichlorvos, Diflubenzuron, Ethion, Ethirimol, Fenamidone, Fenoxaprop-P-ethyl, Fenpropidin, Fipronil, Flufenoxuron, Flusilazole, Hexazinone, Imazaquin, Imidacloprid, Imidaclothiz, Iprobenfos, Isoproturon, Linuron, Metalaxyl, Metazachlor, Methomyl, Metribuzin, Molinate, Oxadiargyl, Oxamyl, Pendimethalin, Pretilachlor, Prochloraz, Prometryn, Pymetrozine, Pyrazosulfuron-ethyl, Simazine, Triadimenol, Triasulfuron, Trichlorfon, Uniconazole). Remaining Pesticides III(Acetochlor, Alachlor, Aldicarb, AmetrynAzoxystrobin, Benalaxyl, Bitertanol, Carbendazim, Carboxin, Cyanazine, Cyprodinil, Difenoconazole, Diuron, Ethoprophos, Fenobucarb, Fludioxonil, Flutolanil, Flutriafol, Hexaconazole, Hexaflumuron, Hexythiazox, Imazalil, Isoprocarb, Ivermectin B1a, Mefenacet, Metolachlor, Napropamide, Oxadiazon, Paclobutrazol, Parathion, Penconazole, Piperonyl butoxide, Propachlor, Propanil, Propazine, Propyzamide, Pyraclostrobin, Pyridaben, Pyriftalid, Pyrimethanil, Rotenone, Simetryn, Spirodiclofen, Spiroxamine (isomer), Teflubenzuron, Terbuthylazine, Thiabendazole, Thiamethoxam, Thidiazuron, Thiobencarb, Thiophanate-methyl, Triadimefon, Pirimiphos-methyl, Edifenphos). **(B)** Mix Standard of GC-MS Chromatograms. Total ion chromatogram (TIC) showing the well-resolved peaks of targeted pesticides involved Cyfluthrin, Cypermethrin, Fenpropathrin, Fenvalerate, Flucythrinate, Lambda-cyhalothrin, Permethrin, Esfenvalerate. **(C)** Typical Real Sample Detection of GC-MS or LC-MS Chromatograms. Total ion chromatogram (TIC) showing the well-resolved peaks of targeted pesticides involved Glufosinate and Glyphosat (LC-MS Chromatogram), Diquat and Paraquat (LC-MS Chromatogram), Lambda-cyhalothrin (GC-MS Chromatogram), Dichlorvos (LC-MS Chromatogram).

To ensure the analytical precision of pesticide detection within the cohort, we validated the performance of our system. High-resolution chromatograms were obtained for both mixed standards (Figures 1A,B). And real biological samples (Figure 1C), with all target compounds showing distinct peak separation and stable retention times.

### 3.2 Clinical characteristics

#### 3.2.1 Distribution of clinical data of the study population by quarter for detected pesticides

Table 1 summarizes the main clinical characteristics of the study participants. Among the 1,641 individuals included in the analysis, 552 (33.6%) had no detectable levels of the target pesticide compounds. There was no significant quarterly variation in the age distribution of the pesticide-exposed population (p = 0.833). The overall male-to-female ratio was nearly balanced at 846:795 (51.6% vs. 48.4%), yet a significant asymmetry in gender distribution among positive cases across quarters was observed (p < 0.001), with males predominating in the second quarter (177/353) and third quarter (187/322).

Among detected pesticides, herbicides exhibited the highest overall detection rate. These included acetochlor, alachlor, ametryn, atrazine, butralin, cinosulfuron, cyanazine, diquat, diuron, glufosinate, glyphosate, hexazinone, metolachlor, paraquat, pendimethalin, piperonyl butoxide, prometryn, propazine, simazine, and simetryn. Significant seasonal heterogeneity in detection rates was evident. Paraquat was detected in 128 out of 373 samples (34.3%), with median concentrations in the third quarter reaching 7,093 μg/L (interquartile range: 1,073–25,654 μg/L), representing a 1.5- to 5-fold increase compared to other quarters. Diquat was identified in 194 of 439 samples (44.2%), peaking in the second quarter with a median concentration of 1,242 μg/L (IQR: 85.66–7,340 μg/L). Among the 292 individuals screened for glyphosate, 144 tested positive, yielding a compound-specific detection frequency of 49.32%. However, when normalized against the entire study population (N = 1641), the cohort-wide prevalence for glyphosate remains at 8.77%, with maximum levels observed in the third quarter (median: 3,725 μg/L; IQR: 268.4–12,750 μg/L). Glufosinate was detected in 76 of 224 samples (33.9%); although showing significant seasonal variation (p < 0.001), its absolute concentration remained lower than that of other herbicides. Among less frequently detected herbicides, acetochlor (48/96, 50%) and atrazine (35/140, 25%) demonstrated moderate detection rates, while diuron and simazine—despite very low overall detection (<2%)—were exclusively identified in the fourth quarter.

**FIGURE 2:**
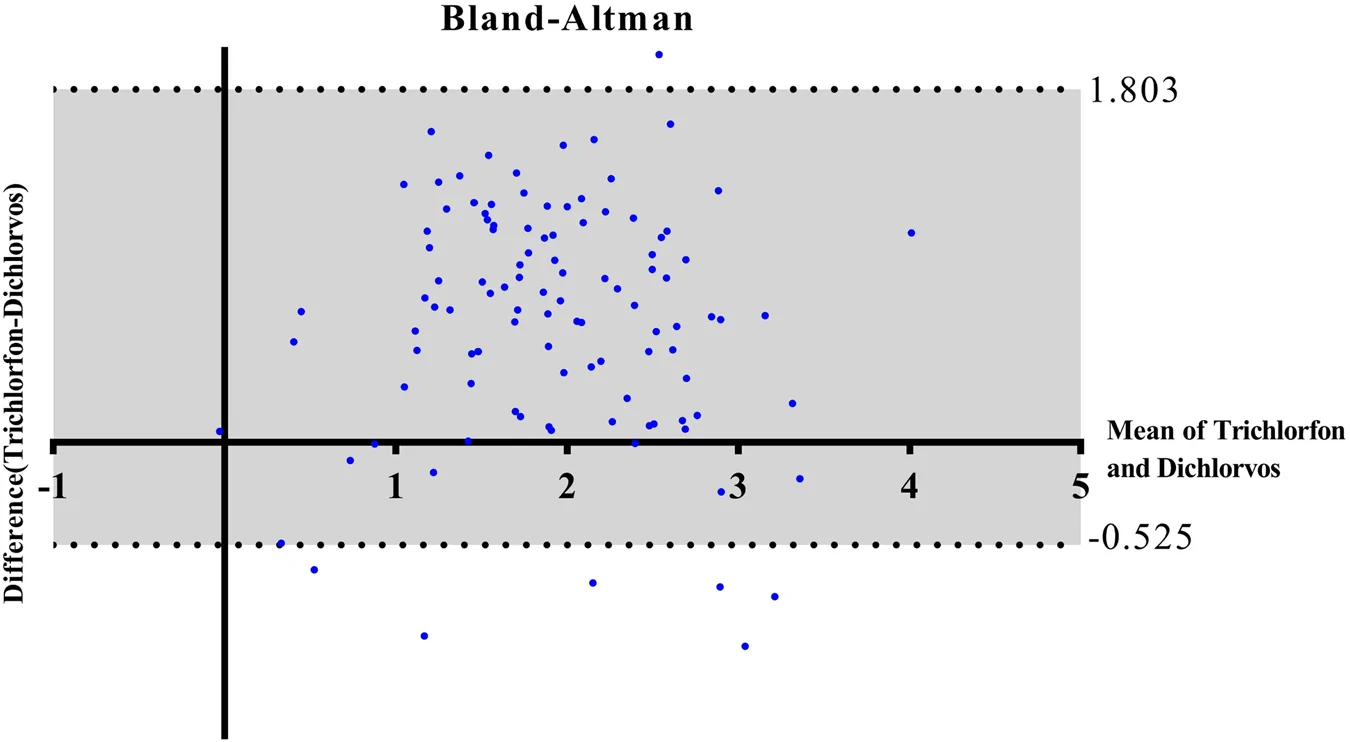
Difference of log10-transformed concentrations between trichlorfon and dichlorvos. The figure reveals the disparities in log_10_-transformed concentration distributions between trichlorfon and dichlorvos. The results demonstrate significant variability in their exposure levels, reflecting the characteristics of congeneric exposure and *in vivo* biotransformation of trichlorfon and dichlorvos in real-world settings.

Insecticides had the second-highest overall positive rate. Detected compounds included acetamiprid, avermectin, bifenthrin, buprofezin, carbofuran, chlorfenapyr, chlorpyrifos, cyfluthrin, cypermethrin, deltamethrin, dichlorvos, dimethoate, esfenvalerate, fenobucarb, fenpropathrin, fenvalerate, fipronil, imidacloprid, isofenphos-methyl, isoprocarb, lambda-cyhalothrin, malathion, methomyl, ometoate, permethrin, phorate, phorate sulfone, phorate sulfoxide, phoxim, pymetrozine, quinalphos, rotenone, sulfotep, thiamethoxam, tralopyril, triazophos, and trichlorfon. Dichlorvos was detected in 118 samples (118/223, 52.9%), whereas its hydrolysis product trichlorfon showed near-universal detection (145/150, 96.67%). Notably, trichlorfon achieved a detection rate of 96.67% (145/150). Correlation analysis revealed that its concentration was strongly synchronized with dichlorvos (Pearson r = 0.7029, p < 0.0001). The concentration differences remain largely within the 95% confidence interval across multiple orders of magnitude (Figure 2), suggesting that the ubiquity of trichlorfon is intrinsically linked to the chemical profile of dichlorvos in this cohort. In contrast, phorate and its metabolites exhibited high detection frequencies (phorate: 27/30, 90.0%; phorate sulfone: 8/11, 72.73%; phorate sulfoxide: 9/12, 75.00%). While phorate sulfone and phorate sulfoxide did not show significant seasonal trends—possibly due to limited sample sizes—phorate itself displayed significant quarterly variation (p = 0.011). Ometoate had a detection rate of 86.96%, with notable differences across quarters and concentration levels spanning four orders of magnitude. Pyrethroid insecticides were generally detected at low rates, with lambda-cyhalothrin showing the highest frequency (38/79, 48.1%) and significant quarterly distribution differences (p = 0.004).

Fungicides were detected at low overall rates and included amitraz, azoxystrobin, carbendazim, difenoconazole, fludioxonil, metalaxyl, prochloraz, pyraclostrobin, pyridaben, tebuconazole, thiophanate-methyl, triadimenol, and triforine. Additionally, the acaricide pyridaben was detected infrequently. Seasonal patterns in pesticide detection varied by chemical class. Herbicides exhibited both qualitative and quantitative seasonal shifts in detection rates and concentrations, whereas insecticides displayed more compound-specific temporal distribution patterns. While certain pesticides, such as isofenphos-methyl (n = 3) and sulfotep (n = 1), these statistics are uninformative due to small positive sample sizes and should be interpreted as potential point-source exposures rather than representative population trends.

#### 3.2.2 Age distribution of the exposed population

**FIGURE 3:**
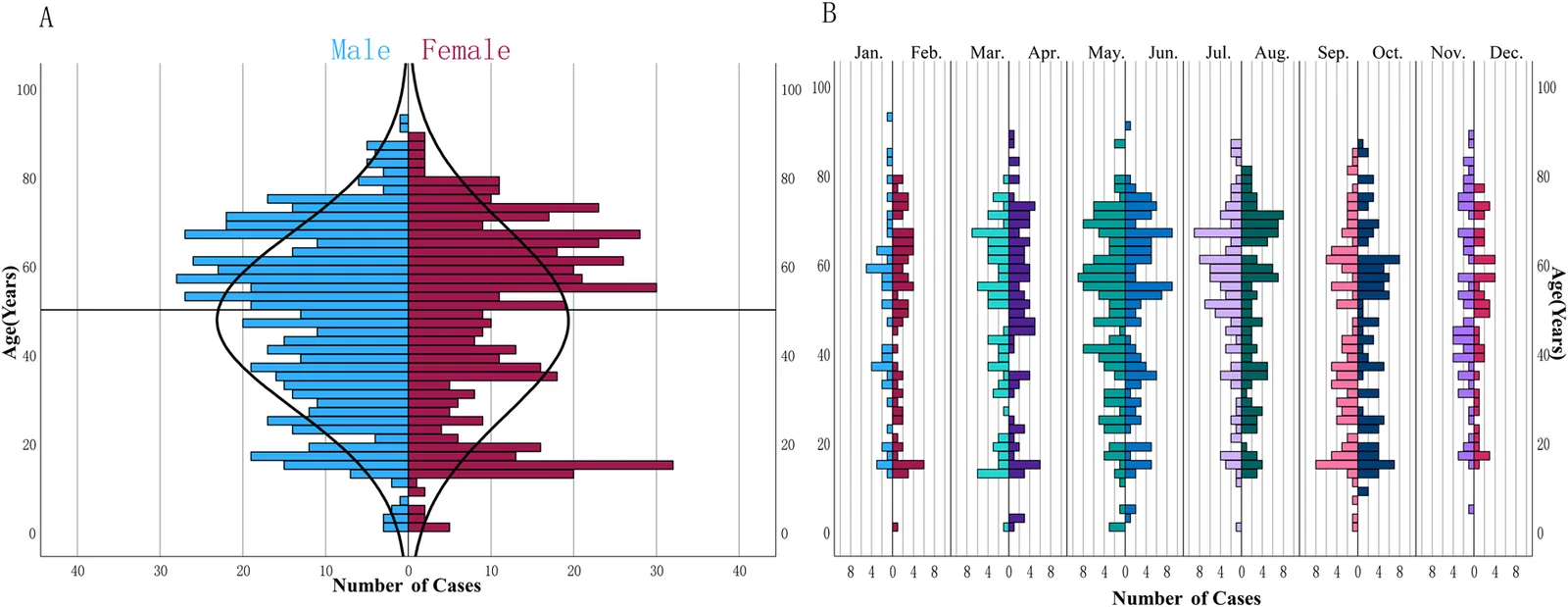
Interaction of gender and month with age in pesticide-exposed populations. **(A)** Gender-age interaction, where the y-axis represents age in years and the x-axis shows case frequency. Blue and red distributions denote male and female exposures, respectively, with black curves representing normal distribution fits. **(B)** Month-age interaction, using the same y-axis for age and x-axis for case frequency, while the top x-axis indicates months (January-December). This panel highlights monthly variations in exposure patterns, with peak frequencies observed during agricultural active seasons (May-August). Both panels illustrate bimodal age distributions, with a primary peak in the 55–60-year age group and a secondary peak at 15–20 years.

The age distribution of the exposed population exhibited slight differences, with a median age of 47 years for men and 51 years for women, indicating a disparity in age structure (Figure 3A). Both male and female cases clustered around two age peaks: a secondary peak observed at 15–20 years and a primary peak where the female peak occurred approximately 5 years later than that of males. The main male peak was located in the 55–60-year age group, while the female peak spanned 55–65 years. Outside these peaks, the age distribution of female cases remained relatively stable. Significant gender-based disparities were identified in the 18–34 age group, with a higher positivity rate observed among female patients compared to males. Conversely, in the geriatric group (≥65 years), male patients demonstrated a clear predominance. Age-monthly trend analysis revealed a significant seasonal shift in the age profile of the exposed population (Figure 3B). The median age of exposed individuals during the first quarter (January–March) ranged between 60 and 65 years. In the second and third quarters, the number of exposed cases increased substantially and was concentrated among those aged 50–70 years. In the fourth quarter, the monthly number of exposed cases declined progressively until December, potentially reflecting reduced agricultural activity during winter months. Notably, although individuals under 30 years of age constituted a small proportion of the cohort, this group showed an exceptionally high exposure rate to specific pesticides such as paraquat, suggesting that exposure risks among younger individuals should not be overlooked. Herbicide exposure also demonstrated clear age-related differentiation. Positive detections of glyphosate, paraquat, and glufosinate were observed throughout the year, with a concentration peak occurring at 50–60 years of age. Specifically, diquat detections exhibited a distinct clustering within the adolescent subgroup, reaching a prominent peak at 14 years of age (n = 12), followed by 15 years (n = 11) and 18 years (n = 10), exhibiting a significantly higher detection rate compared to other pesticides—this pattern may indicate a prominent role of non-occupational accidental exposure. Dichlorvos and trichlorfon were also consistently detected across the year, forming persistent hotspots in the 50–70-year age range. Beyond the metabolic relationship between dichlorvos and trichlorfon, household pesticide use may contribute to these findings. Additionally, fungicides and low-detection-rate insecticides such as fenvalerate and fipronil showed generally weak signals but exhibited disproportionately elevated detection rates in the 70–80-year-old group, possibly due to heterogeneous exposure sources in this older population (Supplementary Figure S1).

#### 3.2.3 Monthly aggregation of pesticide exposure

**FIGURE 4:**
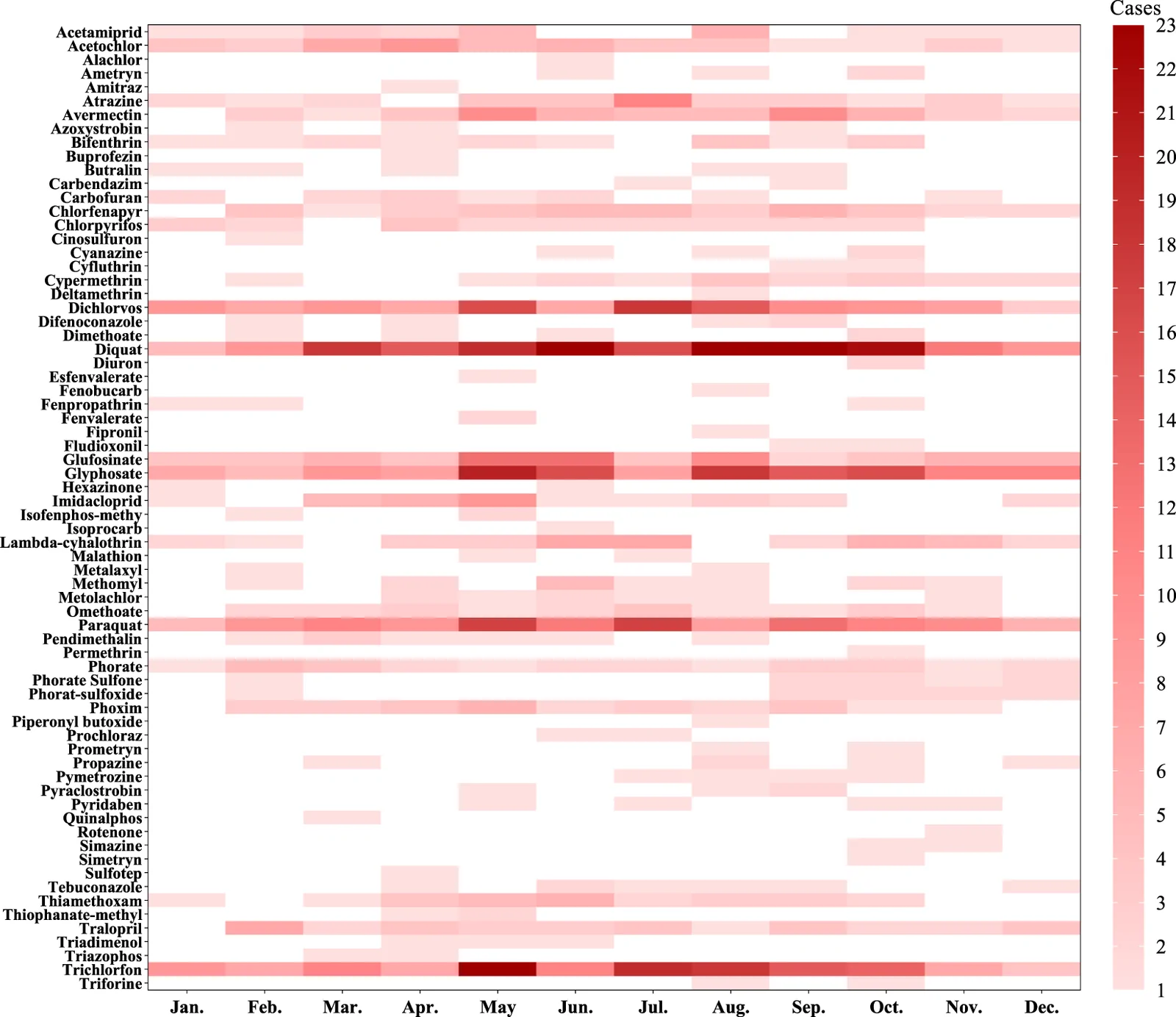
Calendar-month distribution of pesticide exposure in the screened cohort. The X-axis represents months from January to December, while the Y-axis displays the names of detected pesticides. The color gradient (with corresponding legend on the right) indicates the number of positive cases, where darker colors represent higher exposure frequencies of specific pesticides in given months. This highlights seasonal patterns of pesticide exposure within the screened cohort.

The monthly heat map of pesticide exposure uses the number of positive cases as an intensity indicator. Figure 4 visually illustrates pronounced temporal clustering of pesticide detection burdens over the course of the year. The annual peak in positive cases occurs from March to August—the period of heightened agricultural activity (as shown in Figure 3B)—indicating marked seasonal variation in pesticide exposure. The overall peak spans May to August, coinciding with the growth cycles of major crops in Shandong Province, such as wheat and corn. The timing of peaks varies by pesticide class: herbicides peak from June to August, whereas insecticides peak from July to September. Among herbicides, acetochlor, glyphosate, atrazine, diquat, glufosinate, and paraquat are detectable year-round. Acetochlor forms a continuous hotspot from April to May; glyphosate exhibits a bimodal pattern with peaks in May and August; atrazine shows scattered signals with a peak in July; and diquat displays a bimodal distribution, with a primary peak in June and a secondary peak from August to October. Glufosinate and paraquat exposure is highly concentrated between May and August. Among insecticides, avermectin, chlorfenapyr, dichlorvos, tralopril, and trichlorfon are also present throughout the year. Avermectin, chlorfenapyr, and tralopril show low-intensity, relatively uniform distributions annually. Monthly aggregation analysis revealed a striking temporal synchronization in the detection of chlorfenapyr and tralopyril (Figure 4). Chlorfenapyr detections peaked in September (n = 6), with secondary peaks observed in February (n = 4), June (n = 5), and July (n = 5). Correspondingly, tralopyril exhibited a highly aligned distribution, with positive cases recorded in February (n = 7), April (n = 4), July (n = 4), September (n = 4), and December (n = 4). This temporal overlap during both early-year and peak agricultural seasons indicates a significant correlation in their environmental or biological distribution patterns. In contrast, despite trichlorfon being a hydrolysis product of dichlorvos, their temporal patterns differ markedly: trichlorfon peaks in May, while dichlorvos peaks in July. Lambda-cyhalothrin forms a continuous hotspot from July to August, with its detection density potentially influenced by concurrent temperature conditions. The fungicide difenoconazole shows an isolated high signal in September. See Figure 4.

### 3.3 Distribution characteristics of multiple pesticide co-exposure

**FIGURE 5:**
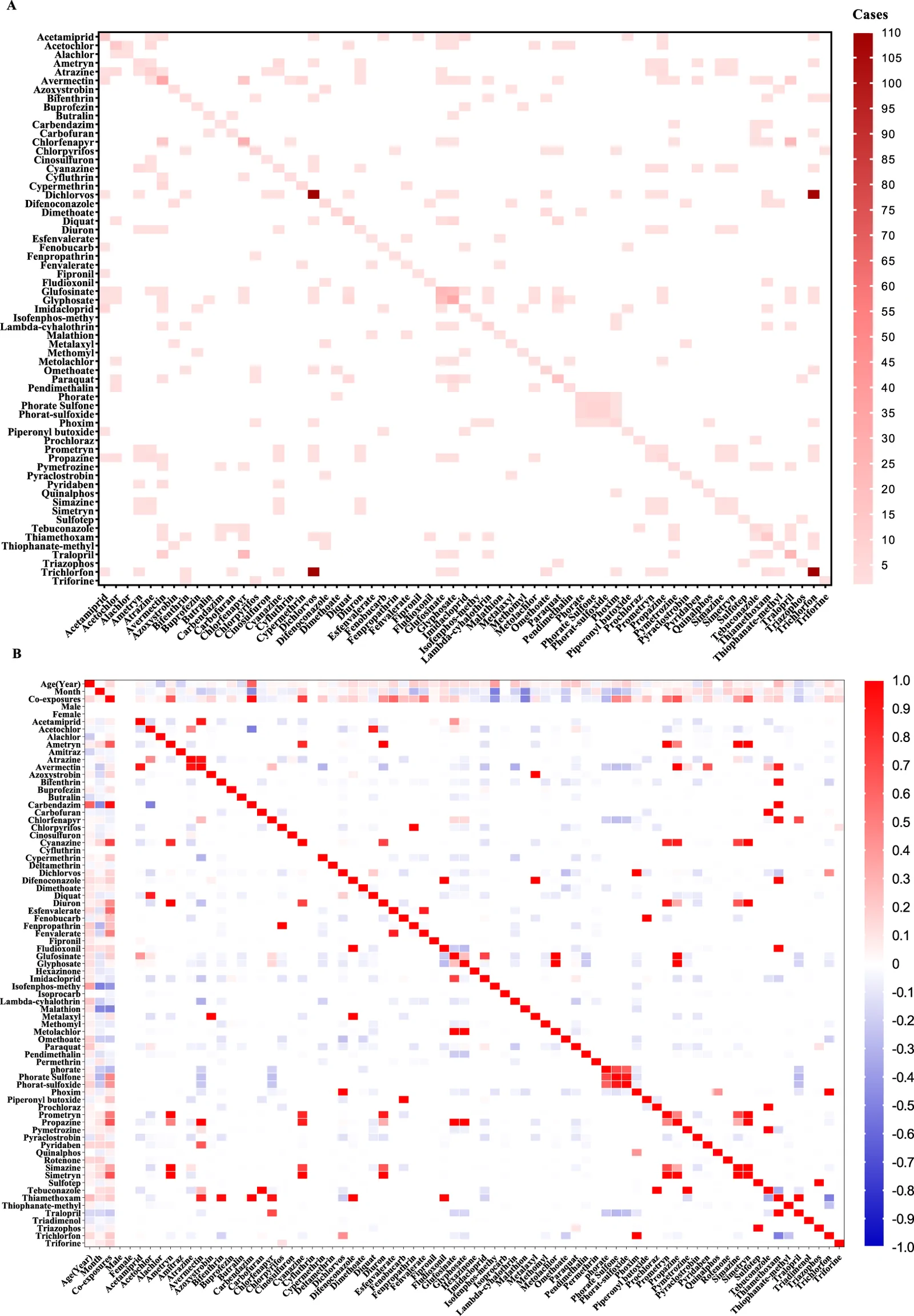
**(A)** Matrix of pesticide co-exposures in the screened cohort. Both axes display detected pesticide compounds, with diagonal elements representing single-pesticide detection frequencies. Color intensity (legend at right) corresponds to the number of cases with concurrent detection of pesticide pairs, where darker shades indicate higher co-exposure frequencies and elevated potential for synergistic health risks. This representation highlights predominant co-exposure clusters, particularly among herbicide combinations (glyphosate-glufosinate) and organophosphate insecticide networks (dichlorvos-trichlorfon-phorate metabolites). **(B)** Correlation matrix of pesticide concentration of co-exposure patterns in the screened cohort. The color gradient represents Pearson correlation coefficients (r) between pesticide concentrations, ranging from −1 (blue) to 1 (red), with non-significant correlations (p ≥ 0.0016 after Bonferroni correction for multiple comparisons) masked in white. Circle size is proportional to the absolute correlation coefficient magnitude. Only statistically significant positive correlations (r ≥ 0.5, p < 0.0016 after Bonferroni correction) are labeled with their correlation coefficients. This visualization reveals significant co-exposure patterns and potential common sources or application practices of pesticide mixtures in the environmental samples.

Figure 5A presents the frequency matrix of pesticide co-exposure, and the corresponding Pearson correlation analysis further reveals statistical dependencies among pesticide detections (Figure 5B; Table 2), uncovering significant co-occurrence patterns. The most frequent co-detection was observed between dichlorvos and trichlorfon (n = 108, r = 0.4396, p < 0.0016), followed by tralopril and chlorfenapyr (n = 23), as well as combinations of glyphosate with glufosinate (n = 20) and paraquat (n = 6). After Bonferroni correction for multiple comparisons (significance threshold p < 0.0016), a total of 38 correlation pairs were identified, revealing distinct co-exposure clusters. (I) Pyrethroid pesticides exhibited complex correlation patterns, although the statistical robustness of certain associations was constrained by sparse data: fenvalerate and esfenvalerate showed a strong correlation (n = 1, r = 0.8834, p < 0.0016), while lambda-cyhalothrin and bifenthrin, despite a high calculated r-value (r = 0.8723, p < 0.0016), were co-detected in only one sample (n = 1), necessitating extreme caution in its ecological interpretation. (II) Glyphosate-related co-exposures formed significant correlations with several pesticides, suggesting specific agricultural combinations: the most notable associations included glufosinate (r = 0.2540, p < 0.0016) and metolachlor (r = 0.9996, p < 0.0016). Among the 38 significant pairs, four of these (carbendazim–thiamethoxam and piperonyl butoxide–fenobucarb, both n = 1) were based on ≤5 co-positive samples. Although statistically significant (p < 0.0016), the small sample size limits their ecological interpretability. In contrast, robust associations such as dichlorvos–trichlorfon (n = 108, r = 0.4396, p < 0.0016) and chlorfenapyr–tralapril (n = 23, r = 0.7077, p < 0.0016) demonstrate significantly greater statistical robustness. Notably, certain fungicides and insecticides exhibited cross-category co-detection patterns, such as azoxystrobin with difenoconazole, reflecting their common use in combination formulations for enhanced fungal control (Devi et al., 2020). Pyridaben, the only detected acaricide and a known mitochondrial electron transport inhibitor (METI) targeting Complex I, displayed prominent cross-category co-occurrence with the insecticide avermectin (n = 3, r = 0.6625) (Cloyd et al., 2010; Wang et al., 2023). However, it is crucial to clarify that avermectin acts as an agonist of glutamate-gated chloride channels (GluCls) and not as a METI (Zhang et al., 2020). Therefore, the observed association does not imply target site overlap at the mitochondrial level. Instead, the potential synergistic effects between pyridaben and avermectin likely arise from complementary physiological impacts—disrupting both energy metabolism and neuromuscular transmission—or from toxicokinetic interactions involving metabolic detoxification pathways, suggesting potential target site overlap and synergistic effects (Adesanya et al., 2020).

**TABLE 2:** Statistically significant co-exposure pairs and correlation parameters in the screened cohort.

Pesticide A	Pesticide B	Detection count (A)	Detection count (B)	Co-detection Count	Pearson r (untransformed concentrations)	P-value (untransformed concentrations)
Acetamiprid	Avermectin	21	55	4*	0.9169	0.0037
Acetamiprid	Imidacloprid	21	30	7	−0.0131	0.8263
Acetochlor	Atrazine	48	35	4*	0.4558	0.1014
Acetochlor	Pendimethalin	48	8	3*	−0.0338	0.7370
Ametryn	Cyanazine	4	4	4*	0.8337	0.0000
Atrazine	Propazine	35	5	3*	−0.0718	0.8154
Atrazine	Diuron	35	2	1*	−0.0101	0.8651
Atrazine	Simazine	35	2	2*	0.1130	0.0579
Avermectin	Chlorfenapyr	55	39	15	0.2590	0.2444
Avermectin	Cypermethrin	55	18	5	−0.2969	0.4751
Avermectin	Pyridaben	55	4	3*	0.6625	0.2229
Avermectin	Tralopril	55	36	10	−0.0777	0.7667
Azoxystrobin	Difenoconazole	3	5	3*	0.0539	0.5917
Bifenthrin	Lambda-cyhalothrin	16	38	1*	−0.0641	0.6355
Carbendazim	Thiamethoxam	2	27	1*	1.0000	0.0000
Chlorfenapyr	Thiamethoxam	39	27	3*	0.9316	0.0683
Chlorfenapyr	Tralopril	39	36	23	0.7077	0.0000
Dichlorvos	Omethoate	118	20	3*	−0.2546	0.7453
Dichlorvos	Trichlorfon	118	145	108	0.4396	0.0000
Difenoconazole	Metalaxyl	5	2	2*	0.9910	0.0000
Difenoconazole	Thiamethoxam	5	27	1*	0.9814	0.0000
Difenoconazole	Fludioxonil	5	2	1*	0.9938	0.0000
Diquat	Glyphosate	194	144	5	−0.0292	0.7352
Diquat	Paraquat	194	128	5	−0.0454	0.3623
Esfenvalerate	Fenvalerate	1	2	1*	0.8834	0.0000
Fenobucarb	Imidacloprid	1	30	1*	0.0183	0.9599
Fludioxonil	Thiamethoxam	2	27	2*	0.9798	0.0000
Glufosinate	Paraquat	76	128	1*	−0.0250	0.7720
Glyphosate	Glufosinate	144	76	20	0.2540	0.0000
Glyphosate	Metolachlor	144	8	2 *	0.9996	0.0000
Glyphosate	Paraquat	144	128	6	0.0236	0.7847
Omethoate	Trichlorfon	20	145	3*	−0.1889	0.8110
Paraquat	Triazophos	128	2	1*	0.1086	0.5896
Phorate	Phorate-sulfone	27	8	6	0.7205	0.0124
Phorate	Phorat-sulfoxide	27	9	6	0.6236	0.0403
Phorate-sulfone	Phorat-sulfoxide	8	9	8	0.9089	0.0000
Pymetrozine	Thiamethoxam	4	27	2*	−0.1488	0.7501
Thiamethoxam	Trichlorfon	27	145	3*	−0.5059	0.4940

N: Total number of participants tested for the specific pesticide; Co-detection Count: Number of cases with concurrent positive results for both Pesticide A and Pesticide B. Pearson r Pearson correlation coefficient calculated based on pesticide concentrations. P-value (Adj.): Significance level adjusted using Bonferroni correction to control the family-wise error rate; the significance threshold was set at P < 0.0016 (0.05/32). * The statistical significance threshold within this dataset, their ecological interpretability and generalizability are constrained by limited sample sizes and should be interpreted with caution.

### 3.4 Log concentration distribution of pesticides

**FIGURE 6:**
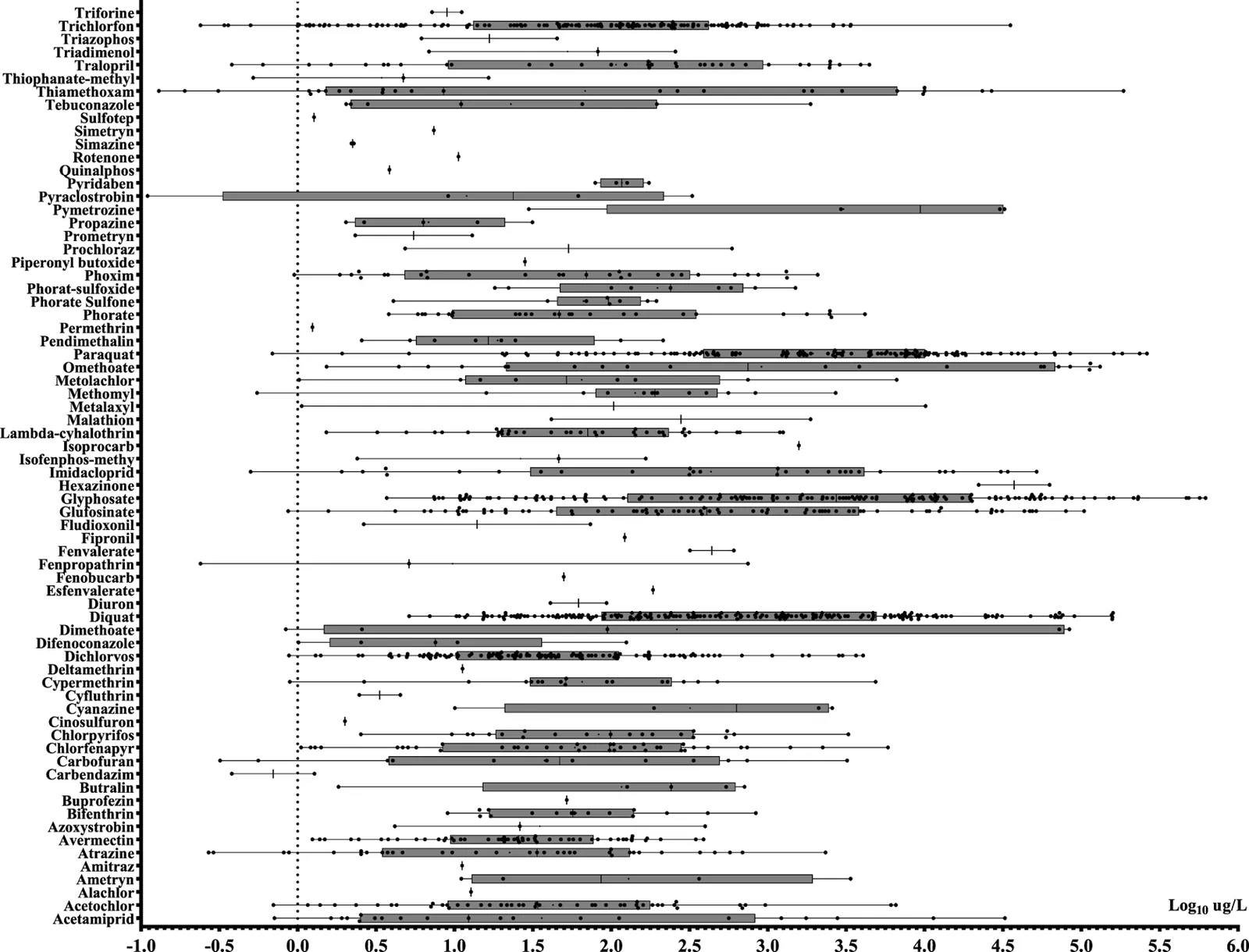
Distribution of log10-transformed pesticide concentrations in the screened cohort. The x-axis represents log10-transformed concentration values (μg/L) of detected pesticides, while the Y-axis displays the names of detected pesticides. Each dot represents a single measurement from a participant, with horizontal box plots depicting the interquartile range (IQR), median (central line), and minimum-maximum values for each pesticide. The vertical dashed line at x = 0 serves as a reference point corresponding to 1 μg/L concentration. This visualization reveals substantial variability in exposure levels across compounds, with organophosphates (trichlorfon, chlorpyrifos) and herbicides (glyphosate, paraquat) showing the highest median concentrations and widest distribution ranges in the cohort.

All pesticide concentrations were log_10_-transformed and exhibited marked heterogeneity. The log-concentration distribution illustrates substantial variation in exposure levels across several orders of magnitude (Figure 6). However, it should be noted that the positive sample sizes for these two compounds are extremely small (n = 3 for isofenphos-methyl; n = 1 for sulfotep), and thus these values should be interpreted with explicit caution as they may not represent broader population trends. Thiamethoxam showed the widest concentration range, with a median of 1.89 log_10_ μg/L (IQR: 1.15–2.63). In contrast, glufosinate had a lower median concentration of 1.58 log_10_ μg/L (IQR: 0.72–2.34), significantly below that of other herbicides such as glyphosate (median: 3.42; IQR: 2.87–4.15). Acetochlor and atrazine exhibited moderate concentration levels, with medians of 1.76 and 2.31 log_10_ μg/L, respectively. Low-frequency herbicides—including ametryn, prometryn, propazine, simazine, simetryn, hexazinone, cinosulfuron, cyanazine, butralin, pendimethalin, metolachlor, diuron, alachlor, and piperonyl butoxide—all had median concentrations below 2.5 log_10_ μg/L. Insecticides displayed diverse exposure profiles: chlorpyrifos had a median log concentration of 3.08 (IQR: 2.44–3.85), while isofenphos-methyl and sulfotep reached notably high levels (4.89 and 4.71 log_10_ μg/L, respectively), suggesting potential point-source exposure risks (Figure 6). Pyrethroids were generally present at low doses: lambda-cyhalothrin had a median concentration of 1.76 log_10_ μg/L (IQR: 0.98–2.45), and the median concentrations of bifenthrin, cyfluthrin, cypermethrin, deltamethrin, fenpropathrin, fenvalerate, esfenvalerate, permethrin, fipronil, chlorfenapyr, and tralopril were all below 2.0 log_10_ μg/L. The median concentrations of trichlorfon, dichlorvos, and phorate were clustered in the medium-to-low range. Among phorate metabolites, phorate sulfoxide exhibited higher concentrations than both its parent compound and phorate sulfone. Acetamiprid and imidacloprid showed similar concentration distributions, with medians of 2.41 log_10_ μg/L (IQR: 1.73–3.16) and 2.18 log_10_ μg/L (IQR: 1.39–2.96), respectively. Overall, fungicide concentrations were generally lower than those of herbicides and insecticides. Azoxystrobin, fludioxonil, amitraz, carbendazim, thiophanate-methyl, triforine, triadimenol, difenoconazole, and prochloraz all remained below 2.0 log_10_ μg/L (Figure 6). Tebuconazole had a median concentration of 1.89 log_10_ μg/L (IQR: 1.15–2.63). Pyraclostrobin, metalaxyl, and pyridaben had concentrations below 2.0 log_10_ μg/L. Pyridaben, the only acaricide detected, had a concentration of 1.86 log_10_ μg/L (IQR: 1.12–2.74). Although the detection rate of specific-purpose pesticides was lower than that of broad-spectrum insecticides, different pesticide categories exhibited significantly distinct exposure patterns. Broad-spectrum herbicides and insecticides showed high variability in concentrations, indicating diverse environmental exposure pathways, whereas specific-purpose pesticides—such as certain fungicides—displayed relatively stable exposure levels, likely attributable to consistent occupational exposure settings.

## 4 Discussion

While previous investigations in Shandong Province have largely addressed endpoint residue monitoring in agricultural products (e.g., vegetables) or single-agent risk assessments (Valcke et al., 2017), this study offers a more comprehensive perspective through the systematic characterization of multidimensional heterogeneity in pesticide co-exposure. By integrating screening of 159 compounds across a large clinical cohort (n = 1,641), we uniquely reveal interconnected patterns across temporal, demographic, and chemical dimensions. Herbicides were the predominant class of detected substances, with a positive detection rate of 49.3%. Notably, glyphosate and paraquat both reached peak concentrations in the third quarter, with median levels of 3,725 μg/L and 7,093 μg/L, respectively—1.5 to 5 times higher than those observed in other quarters. To rigorously characterize the overall heterogeneity without allowing these extreme data points to over-dominate the analysis, we implemented a log_10_ transformation strategy. Regarding the etiology of these exceptionally high concentrations. This temporal pattern aligns with findings from passive atmospheric sampling in the North China Plain reported by Zhao et al. (2023), supporting the role of regional agricultural practices as a key driver of seasonal exposure peaks, particularly among core labor ages (55–60 years). However, the exceptional magnitude of these peaks, combined with the secondary peak identified in adolescents (15–20 years), suggests that such extreme data points likely integrate intentional acute exposure (e.g., self-poisoning) alongside occupational risks (Pujo et al., 2024). Specifically, our findings link clinical internal exposure peaks (May–August) with regional atmospheric monitoring data, define age-specific bimodal distribution patterns, and establish structural co-exposure networks that reflect the regional cropping systems, thereby providing a new paradigm for population-level pesticide exposure assessment in this intensive agricultural region.

The age distribution of pesticide exposure exhibits a distinct bimodal pattern. The primary peak (55–60 years) reflects the occupational profile of the core agricultural workforce, whose intensive spraying activities during the May–August period (Q3) drive a significant seasonal demographic shift. As illustrated in Figure 4, the overall exposure burden reaches its annual peak from May to August, coinciding with the growth cycles and pesticide application windows of major crops in the North China Plain, such as wheat and corn. During this peak season (Q3), middle-aged individuals (aged 40–54) become the most directly exposed group due to intensive field labor, shifting the median age downward to 48–50 years. Conversely, the higher median age of 58 in Q1 reflects a shift toward older populations during the non-spraying season, where positive detections are primarily driven by the long-term accumulation of low-dose environmental residues or persistent household hotspots (e.g., dichlorvos and trichlorfon) identified in the 50–70-year age range in Supplementary Figure S1 (Cha et al., 2020). In contrast, the secondary peak among adolescents (15–20 years), particularly the notable detection of diquat at ages 14–18, more likely corresponds to non-occupational intentional exposure rather than school-age labor (dos Santos et al., 2020). This aligns with findings that the easy accessibility of pesticides in rural households significantly facilitates impulsive self-harm incidents (Wang et al., 2020; Weerasinghe et al., 2020a). The specific relevance of diquat, which has seen increased usage following the paraquat ban, further supports this hypothesis due to its high lethality and association with severe toxic encephalopathy and multi-organ failure (Alhassan and Aljahdali, 2024; Liu and Jiang, 2025; Weerasinghe et al., 2020b). Thus, the bimodal distribution reflects a synthesis of long-term occupational exposure in older adults and intentional self-poisoning among youth populations, the latter primarily driven by mental health factors and lethal means accessibility rather than agricultural labor patterns (Kootbodien et al., 2021; Wang et al., 2020).

Gender-stratified analysis revealed higher positive rates among men compared to women in the second and third quarters. However, young women (aged 18–34) showed a higher positive rate than their male counterparts, suggesting additional exposure sources beyond agriculture, such as household pesticide use in dormitories or rental housing—a pattern consistent with urban exposure profiles documented by Peng et al. (2020). Age-based clustering indicated that individuals aged 40–54 accounted for 58.3% of all positive cases, reflecting their central role in agricultural labor. This is further supported by WHO reports indicating that agricultural workers in developing countries face significantly higher pesticide exposure risks than the general population, attributable to frequent occupational contact and inadequate protective measures (WHO, 2025; Zúñiga-Venegas et al., 2025). Furthermore, biomonitoring studies have corroborated that pesticide residue levels or the prevalence of abnormal biomarkers in agricultural workers are substantially elevated compared to non-agricultural control groups (Guytingco et al., 2018; Leili et al., 2022). As a major agricultural nation, China’s rural populations—particularly middle-aged and elderly farmers—are frequently exposed to pesticides and often lack adequate protective measures (Li et al., 2023; Sun et al., 2019; Tudi et al., 2022), increasing susceptibility to accidental poisoning (Gunnell et al., 2007; London and Bailie, 2001).

Notably, dichlorvos exposure hotspots were identified in the 18–34 age group. Given its widespread use in fumigating stored fruits and vegetables, this finding suggests potential spillover exposure from post-harvest agricultural supply chains into urban youth populations. A similar trend has been observed by Nguyen in Hanoi’s vegetable wholesale markets, Vietnam (Nguyen Dang Giang et al., 2022). Regarding young adults (18–34 years), identified dichlorvos hotspots are speculatively linked to post-harvest supply chain spillover. Dichlorvos is recognized for its utility in protecting stored agricultural products (Kong et al., 2016). The temporal divergence observed in our data—where dichlorvos peaks in July while its hydrolysis product trichlorfon peaks in May—points toward potential independent external exposure sources. Residues of field-applied pesticides can persist and transform during storage, posing exposure risks to workers involved in labor-intensive harvesting, transportation, and processing stages (Kong et al., 2016; Soupene et al., 2022). Such occupational risks are often exacerbated by inadequate safety standards and protective equipment in small-scale storage facilities, particularly for inexperienced younger workers (Ye et al., 2013). While the direct link between supply chain mechanisms and dichlorvos poisoning remains speculative, the convergence of occupational labor patterns, storage-phase pesticide use, and insufficient safety measures provides a plausible basis for this hypothesis, warranting future detailed exposure assessments in post-harvest facilities (Gois et al., 2023; Ismail et al., 2021; Kong et al., 2016).

The co-occurrence network provides the first evidence of a triangular synergy among herbicides and cross-category synergistic interactions. The co-detection of glyphosate and glufosinate reflects the widespread practice of “total vegetation control” in certain agricultural regions of China. From a public health perspective, these identified clusters—notably the herbicide pairings and the organophosphate network—offer critical application scenarios for transitioning risk assessment from single-agent limits toward synergistic toxicity paradigms, as such ‘pesticide cocktails’ have been shown to exert significantly greater neurotoxicity than individual constituents (Oltramare et al., 2023). The co-occurrence of dichlorvos and trichlorfon, with trichlorfon concentrations peaking 2 months after dichlorvos, aligns with the hydrolysis kinetics of organophosphate pesticides under warm and humid conditions (Chen et al., 2022; Lian et al., 2021). While this pattern confirms intensive agricultural activity as the primary driver, the dichlorvos hotspots observed in younger, non-occupational populations further suggest an independent ‘post-harvest supply chain’ exposure route, likely arising from precursor transformation during storage or processing (Kong et al., 2016). Similarly, the concurrent detection of pyridaben, chlorfenapyr, and avermectin suggests a strategic combination of pesticides with overlapping targets and synergistic effects, consistent with findings reported by Yuan et al. (2025). Log-transformed pesticide concentration distributions revealed significant differences in variability across categories. Herbicides (e.g., glyphosate and paraquat) and some broad-spectrum insecticides (e.g., chlorfenapyr and tralopril) exhibited highly right-skewed distributions, indicating sporadic but intense exposure events. This distribution profile, coupled with the bimodal age distribution, highlights the study’s dual significance: informing the need for enhanced occupational protection for elderly farmers during peak seasons (Thredgold et al., 2019), and providing a data-driven basis for restricting point-of-sale access to mitigate impulsive self-poisoning incidents among rural youth (Wang et al., 2020). In contrast, specific-purpose fungicides (e.g., tebuconazole) and plant growth regulators (e.g., cinosulfuron) showed more concentrated distribution patterns, consistent with FAO-reported exposure levels (WHO, 2025).

In conclusion, pesticide co-exposure in eastern agricultural regions of China is characterized by multi-pesticide synergy, where seasonal patterns, crop management cycles, occupational practices, gender, age, and exposure scenarios collectively define vulnerable populations. This model moves beyond the traditional “single pesticide–single pathway” risk assessment framework, uncovering the complex drivers of exposure and the cumulative risks associated with multiple active ingredients (Tang et al., 2021). Within the co-occurrence network, the metabolic sequence phorate → sulfoxide → sulfone does not merely represent sequential transformation but may imply non-additive health impacts. Our clinical cohort identified co-occurrence within this chain, with concentrations exhibiting correlation (Pearson r ranging from 0.62 to 0.91), Metabolite concentrations—particularly phorate sulfoxide—frequently exceeded those of the parent compound. Although current “exposure-effect” models focus on individual compounds, this approach may underestimate the total biological burden, as organophosphate oxidation products often possess greater biological stability and enhanced potency in acetylcholinesterase inhibition compared to parent compounds (Alhassan and Aljahdali, 2024). Current challenges in pesticide risk and residue assessment highlighted by FAO/WHO are closely linked to food safety, sustainable agriculture, and international trade (WHO, 2025). Domestically, resistance management-driven mixing practices underscore an urgent need for regionally coordinated exposure limits (Buscaroli et al., 2021). Consequently, the potential for systematic underestimation of cumulative health risks—particularly neurological or renal impairment—cannot be entirely ruled out in models focusing on single active ingredients (Ismail et al., 2021). Future iterations of this surveillance framework should aim to integrate “Total Toxicity Equivalents” by assigning weighted factors to critical metabolites based on their relative potency factors (RPF), thereby enhancing the predictive sensitivity for early-stage biochemical injury. Crucially, the identified ‘low-dose uniformity’ of fungicides indicates that risk assessment must transcend peak-intensity analysis to address this persistent physiological pressure background. Although fungicide concentrations may remain below the thresholds seen with acute herbicide events, they constitute a pervasive ‘background load’ that facilitates insidious, irreversible organ damage over time, particularly during critical developmental windows for vulnerable sub-populations (Ismail et al., 2021). Given the challenges in monitoring these near-LOD residues and their significant cumulative contribution to the total chronic health burden (Valcke et al., 2017), establishing a comprehensive, long-term Toxicovigilance framework for multi-class mixture exposures is now a public health imperative (Alcala et al., 2022; Pujo et al., 2024). Furthermore, dichlorvos hotspots in younger populations suggest that the “post-harvest agricultural supply chain” has emerged as a new exposure pathway, echoing Marc’s concerns regarding urban food safety (Godswill Ntsefong, 2025).

Our findings suggests that tailored protective measures should prioritize middle-aged and elderly male agricultural workers (specifically those aged 40–55 and >65 years), particularly during the third quarter which coincides with peak agricultural activities in the North China Plain. These demographic hotspots align with the age groups most heavily involved in intensive pesticide application and field management within regional cropping systems Nevertheless, this study has several limitations. Data from a single center may limit generalizability to the broader provincial population; the cross-sectional design precludes causal inference between pesticide exposure and health outcomes; and the absence of environmental media (e.g., water, soil) data hampers precise source attribution. Future research should integrate environmental and biological monitoring data and employ prospective cohort designs to evaluate the long-term health effects of specific pesticide mixtures. While our current analysis quantifies the statistical dependencies between specific pesticide pairs using Pearson correlation coefficients, future assessments should move beyond the traditional ‘single pesticide-single pathway’ framework to more accurately capture the cumulative risk of chronic, low-dose mixture exposures. Advanced network-based computational frameworks, such as multiplex network ranking algorithms developed for heterogeneous complex disease analysis, may offer future opportunities for integrating multi-dimensional exposure data—spanning temporal fluctuations, demographic disparities, and chemical synergies (Li et al., 2022). Such methodologies would facilitate a more granular understanding of the biological distribution patterns identified in this cohort, ultimately informing targeted public health policies and precise intervention thresholds.

## Data Availability

The original contributions presented in the study are included in the article/Supplementary Material, further inquiries can be directed to the corresponding authors.

## References

[B1] Adesanya A. W. Cardenas A. Lavine M. D. Walsh D. B. Lavine L. C. Zhu F. (2020). RNA interference of NADPH-Cytochrome P450 reductase increases susceptibilities to multiple acaricides in Tetranychus urticae. Pesticide Biochem. Physiology 165, 104550. 10.1016/j.pestbp.2020.02.016 32359548

[B2] Alcala C. S. Lichtveld M. Y. Wickliffe J. K. Zijlmans W. Shankar A. Rokicki E. (2022). Characterization of urinary pesticide metabolite concentrations of pregnant women in Suriname. TOXICS 10, 679. 10.3390/toxics10110679 36355970 PMC9695383

[B3] Alhassan A. B. Aljahdali M. O. (2024). Behavioural and biochemical responses of freshwater bivalve Anodonta marginata exposed to Dichlorvos. WATER 16, 3572. 10.3390/w16243572

[B4] Antonangeli L. M. Kenzhebekova S. Colosio C. (2023). Neurobehavioral effects of low-dose chronic exposure to insecticides: a review. TOXICS 11, 192. 10.3390/toxics11020192 36851066 PMC9963921

[B5] Supanekar S. P. (2022). Environmental impacts of pesticides - a review. Int. J. Zoological Investigations 8, 847–856. 10.33745/ijzi.2022.v08i02.102

[B6] Bao W. Liu B. Simonsen D. W. Lehmler H.-J. (2020). Association between exposure to pyrethroid insecticides and risk of all-cause and cause-specific mortality in the general US adult population. JAMA Intern. Med. 180, 367–374. 10.1001/jamainternmed.2019.6019 31886824 PMC6990752

[B7] Barrón Cuenca J. Tirado N. Vikström M. Lindh C. H. Stenius U. Leander K. (2020). Pesticide exposure among Bolivian farmers: associations between worker protection and exposure biomarkers. J. Expo. Sci. Environ. Epidemiol. 30, 730–742. 10.1038/s41370-019-0128-3 30787424 PMC8608618

[B8] Beyuo J. Sackey L. Yeboah C. Kayoung P. Y. Koudadje D. (2024). The implications of pesticide residue in food crops on human health: a critical review. Discov. Agric. 2, 123. 10.1007/s44279-024-00141-z

[B9] Buscaroli E. Braschi I. Cirillo C. Fargue-Lelièvre A. Modarelli G. C. Pennisi G. (2021). Reviewing chemical and biological risks in urban agriculture: a comprehensive framework for a food safety assessment of city region food systems. Food control. 126, 108085. 10.1016/j.foodcont.2021.108085 34345121 PMC8080888

[B10] Cha E. S. Chang S. S. Choi Y. Lee W. J. (2020). Trends in pesticide suicide in South Korea, 1983-2014. Epidemiol. PSYCHIATRIC Sci. 29, e25. 10.1017/S2045796019000118 30885286 PMC8063219

[B11] Chen R. Luo X. Liang G. (2022). Hydrolysis of an organophosphorus pesticide: a computational reaction study on triazophos. Theor. Chem. Accounts 141, 62. 10.1007/s00214-022-02925-2

[B12] Cloyd R. Galle C. Keith S. Kemp K. E. (2010). Effect of fungicides and miticides with Mitochondria Electron transport inhibiting activity on the twospotted spider mite, Tetranychus urticae (Acari: tetranychidae). Hortscience 45, 687–689. 10.21273/hortsci.45.4.687

[B13] Commission E. (2024). Analytical Quality Control and Method Validation Procedures for Pesticide Residues Analysis in Food and Feed (SANTE/11312/2021). Brussels: European Commission Directorate-General for Health and Food Safety.

[B14] Devi B. Gupta S. K. Singh G. Prasad P. (2020). Efficacy of new generation fungicides against French bean rust caused by Uromyces appendiculatus. Phytoparasitica 48, 535–543. 10.1007/s12600-020-00820-9

[B15] Dos Santos J. C. P. Valli J. B. Sesse N. S. Mackenzie-Ross S. Zandonade E. Ayres L. R. (2020). Sociodemographic characteristics and exposure patterns of pesticide-related cases reported to a poison service center in Brazil between 2012 and 2016. Archives Of Environ. and Occup. Health 76, 494–503. 10.1080/19338244.2020.1848773 33252014

[B16] Godswill Ntsefong N. (2025). “The rise of urban agriculture and its implications for food systems,” in Worldwide Megatrends in Food Safety and Food Security. Editors Marc R. A. Mureşan C. C. A. Narcisa Postolache. (London: IntechOpen). 10.5772/intechopen.1005284

[B17] Gois M. F. B. Fernández-Pato A. Huss A. Gacesa R. Wijmenga C. Weersma R. K. (2023). Impact of occupational pesticide exposure on the human gut microbiome. Front. Microbiol. 14, 1223120. 10.3389/fmicb.2023.1223120 37637104 PMC10448898

[B18] Gunnell D. Eddleston M. Phillips M. R. Konradsen F. (2007). The global distribution of fatal pesticide self-poisoning: systematic review. BMC Public Health 7, 357. 10.1186/1471-2458-7-357 18154668 PMC2262093

[B19] Guytingco A. Thepaksorn P. Neitzel R. L. (2018). Prevalence of abnormal serum cholinesterase and associated symptoms from pesticide exposure among agricultural workers in the south of Thailand. J. Agromedicine 23, 270–278. 10.1080/1059924X.2018.1470049 30047860

[B20] Ismail A. A. Hendy O. Abdel Rasoul G. Olson J. R. Bonner M. R. Rohlman D. S. (2021). Acute and cumulative effects of repeated exposure to chlorpyrifos on the liver and kidney function among Egyptian adolescents. Toxics 9, 137. 10.3390/toxics9060137 34200920 PMC8230541

[B21] Jallow M. F. A. Awadh D. G. Albaho M. S. Devi V. Y. Thomas B. M. (2017). Pesticide risk behaviors and factors influencing pesticide use among farmers in Kuwait. Sci. Total Environ. 574, 490–498. 10.1016/j.scitotenv.2016.09.085 27644027

[B22] Kong Z. Q. Li M. M. Chen J. Y. Gui Y. J. Bao Y. M. Fan B. (2016). Behavior of field-applied triadimefon, malathion, dichlorvos, and their main metabolites during barley storage and beer processing. FOOD Chem. 211, 679–686. 10.1016/j.foodchem.2016.05.058 27283683

[B23] Kootbodien T. Holtman Z. Asmal L. Joska J. Chiliza B. Smith P. (2021). Organophosphate pesticide exposure as a risk factor for attempted suicide in Cape Town, South Africa: a case-control study. Archives Of Environ. and Occup. Health 77, 789–799. 10.1080/19338244.2021.2018983 34933659

[B24] Leili M. Ghafiuri-Khosroshahi A. Poorolajal J. Samiee F. Smadi M. T. Bahrami A. (2022). Pesticide residues levels as hematological biomarkers—a case study, blood serum of greenhouse workers in the city of Hamadan, Iran. Environ. Sci. Pollut. Res. 29, 38450–38463. 10.1007/s11356-021-17637-6 35080720

[B25] Li X. Xiang J. Wu F. X. Li M. (2022). A dual ranking Algorithm based on the multiplex network for heterogeneous complex disease analysis. IEEE/ACM Trans. Comput. Biol. Bioinforma. 19, 1993–2002. 10.1109/tcbb.2021.3059046 33577455

[B26] Li H. Wang C. Chang W.-Y. Liu H. (2023). Factors affecting Chinese farmers' environment-friendly pesticide application behavior: a meta-analysis. J. Clean. Prod. 409, 137277. 10.1016/j.jclepro.2023.137277

[B27] Li X. Li Q. Xu Q. Huang M. Li W. Wu Q. (2026). Exploration of complex-based probe for pesticide residue detection: specific recognition achieved *via* the synergistic effect of coordination water and supramolecular chains. Food Chem. 503, 147837. 10.1016/j.foodchem.2025.147837 41499899

[B28] Lian L. Jiang B. Xing Y. Zhang N. (2021). Identification of photodegradation product of organophosphorus pesticides and elucidation of transformation mechanism under simulated sunlight irradiation. Ecotoxicol. Environ. Saf. 224, 112655. 10.1016/j.ecoenv.2021.112655 34418856

[B29] Liu Z. Jiang Y. (2025). Toxic encephalopathy induced by diquat poisoning: a case series of three patients. Int. J. Biol. Life Sci. 9, 33–35. 10.54097/bph6v614

[B30] London L. Bailie R. (2001). Challenges for improving surveillance for pesticide poisoning: policy implications for developing countries. Int. J. Epidemiol. 30, 564–570. 10.1093/ije/30.3.564 11416084

[B31] López-Maldonado E. Mahmoud A. Alfarra F. Can-Güven E. Ciner M. N. Guvenc S. Y. (2025). A review on occurrences, challenges and detection methods of pesticides: recent treatment technologies. Int. J. Environ. Sci. Technol. 22, 14771–14815. 10.1007/s13762-025-06640-w

[B32] Nguyen Dang Giang C. Le D. B. C. Nguyen V. H. Hoang T. L. Tran T. V. T. Huynh T. P. L. (2022). Assessment of pesticide use and pesticide residues in vegetables from two provinces in Central Vietnam. PLoS One 17, e0269789. 10.1371/journal.pone.0269789 35696374 PMC9191740

[B33] Oltramare C. Mediouni Z. Shoman Y. Hopf N. B. Graczyk H. Berthet A. (2023). Determinants of pesticide exposure in occupational studies: a meta-analysis. Toxics 11, 623. 10.3390/toxics11070623 37505588 PMC10386710

[B36] Peng F. J. Hardy E. M. Mezzache S. Bourokba N. Palazzi P. Stojiljkovic N. (2020). Exposure to multiclass pesticides among female adult population in two Chinese cities revealed by hair analysis. Environ. Int. 138, 105633. 10.1016/j.envint.2020.105633 32179318

[B37] Pujo J. M. Simon Y. Lontsi Ngoulla G. R. Signaté B. Mutricy R. Frémery A. (2024). Clinical and epidemiological characteristics of severe acute adult poisonings in French Amazonia: urgent need for a toxicovigilance monitoring framework. Toxics 12, 200. 10.3390/toxics12030200 38535933 PMC10975947

[B38] Qin X. Cui H. Zhou Q. (2023). Physisorption behaviors of organochlorine pesticides on the InP(3) monolayer from theoretical Insight. ACS Omega 8, 32168–32175. 10.1021/acsomega.3c04665 37692222 PMC10483652

[B39] Soupene V. A. Casteel C. Nonnenmann M. Rohlman D. S. (2022). Safety measures, pesticide concerns and resources utilized among young adult workers: a brief report. J. Agromedicine 28, 609–614. 10.1080/1059924X.2022.2155748 36469529 PMC10225311

[B40] Sun S. Hu R. Zhang C. Shi G. (2019). Do farmers misuse pesticides in crop production in China? Evidence from a farm household survey. Pest Manag. Sci. 75, 2133–2141. 10.1002/ps.5332 30632284

[B41] Suo C. Zhang L. Liang Q. Chen C. Qiu L. Li Z. (2026). Discovery of farnesal from a wild cucumber landrace enables eco-compatible biopesticide development against aphids. Nat. Commun. 10.1038/s41467-026-72502-9 PMC13319835 42031760

[B42] Tang F. Lenzen M. Mcbratney A. Maggi F. (2021). Risk of pesticide pollution at the global scale. Nat. Geosci. 14, 206–210. 10.1038/s41561-021-00712-5

[B43] Thredgold L. Gaskin S. Quy C. Pisaniello D. (2019). Exposure of agriculture workers to pesticides: the effect of heat on protective glove performance and skin exposure to dichlorvos. Int. J. Environ. Res. PUBLIC HEALTH 16, 4798. 10.3390/ijerph16234798 31795387 PMC6926567

[B44] Tudi M. Li H. Li H. Wang L. Lyu J. Yang L. (2022). Exposure routes and health risks associated with pesticide application. Toxics 10, 335. 10.3390/toxics10060335 35736943 PMC9231402

[B45] Valcke M. Bourgault M. H. Rochette L. Normandin L. Samuel O. Belleville D. (2017). Human health risk assessment on the consumption of fruits and vegetables containing residual pesticides: a cancer and non-cancer risk/benefit perspective. Environ. Int. 108, 63–74. 10.1016/j.envint.2017.07.023 28802169

[B46] Wang B. S. Han L. Wen J. B. Zhang J. Zhu B. L. (2020). Self-poisoning with pesticides in Jiangsu Province, China: a cross-sectional study on 24,602 subjects. BMC PSYCHIATRY 20, 545. 10.1186/s12888-020-02882-9 33225936 PMC7681979

[B47] Wang W. Kuai L. Zhang M. (2023). Chlorfenapyr induces cardiotoxicity by downregulating Dap3 to induce mitochondrial damage. J. Biol. Regul. Homeost. Agents 37, 4861–4871. 10.23812/j.biol.regul.homeost.agents.20233709.473

[B48] Weerasinghe M. Konradsen F. Eddleston M. Pearson M. Jayamanne S. Knipe D. (2020a). Factors associated with purchasing pesticide from shops for intentional self‐poisoning in Sri Lanka. Trop. Med. and Int. Health 25, 1198–1204. 10.1111/tmi.13469 33463883

[B49] Weerasinghe M. Pearson M. Konradsen F. Agampodi S. Sumith J. A. Jayamanne S. (2020b). Emerging pesticides responsible for suicide in rural Sri Lanka following the 2008–2014 pesticide bans. BMC Public Health 20, 780. 10.1186/s12889-020-08871-7 32450831 PMC7249439

[B50] WHO (2025). Pesticide Residues in Food: Report 2024 – Joint FAO/WHO Meeting on Pesticide Residues. Rome. 10.4060/cd5918en

[B51] Wolfe J. Marsit C. (2023). Pyrethroid pesticide exposure and placental effects. Mol. Cell. Endocrinol. 578, 112070. 10.1016/j.mce.2023.112070 37722502 PMC10591723

[B52] Wu K. Chen C. (2025). Spatiotemporal differentiation of fertilizer and pesticide use and its driving factors in the Yangtze River Delta of China: an analysis at the county Scale. Land 14, 1180. 10.3390/land14061180

[B53] Xiangguo F. (2024). Shandong Statistical Yearbook 2023. Beijing: China Statistics Press.

[B54] Ye M. Beach J. Martin J. W. Senthilselvan A. (2013). Occupational pesticide exposures and respiratory health. Int. J. Environ. Res. PUBLIC HEALTH 10, 6442–6471. 10.3390/ijerph10126442 24287863 PMC3881124

[B55] Yuan K. Dai T. Luo B. Chen J. Liu R. Liu X. (2025). Verification of resistance mechanism of mitochondrial electron transport chain complex III inhibitors in Phytophthora sojae through ectopic overexpression. J. Agric. Food Chem. 73, 8876–8885. 10.1021/acs.jafc.5c00747 40176201

[B56] Zhang X. Zhang G. Zhai W. Zhao Z. Wang S. Yi J. (2020). Inhibition of TMEM16A Ca2+-activated cl− channels by avermectins is essential for their anticancer effects. Pharmacol. Res. 156, 104763. 10.1016/j.phrs.2020.104763 32201246

[B57] Zhao M. Wu J. Figueiredo D. M. Zhang Y. Zou Z. Cao Y. (2023). Spatial-temporal distribution and potential risk of pesticides in ambient air in the North China Plain. Environ. Int. 182, 108342. 10.1016/j.envint.2023.108342 38006771

[B58] Zúñiga-Venegas L. Landeros N. Pancetti F. Cortés S. Lucero B. Brito A. M. (2025). Validation of a novel questionnaire for assessing occupational exposure to organophosphate pesticides in Chilean agricultural workers. Front. Toxicol. 7, 1588408. 10.3389/ftox.2025.1588408 40901084 PMC12399619

