# Supplementary material for: Multidimensional characterization of heterogeneity in pesticide co-exposure patterns
Source: Front Chem. 2026 Aug 14;14:1890266. doi: 10.3389/fchem.2026.1890266 (PMC13522405; doi:10.3389/fchem.2026.1890266)
Supplement: Supplementary file 1 [file Image1.pdf]

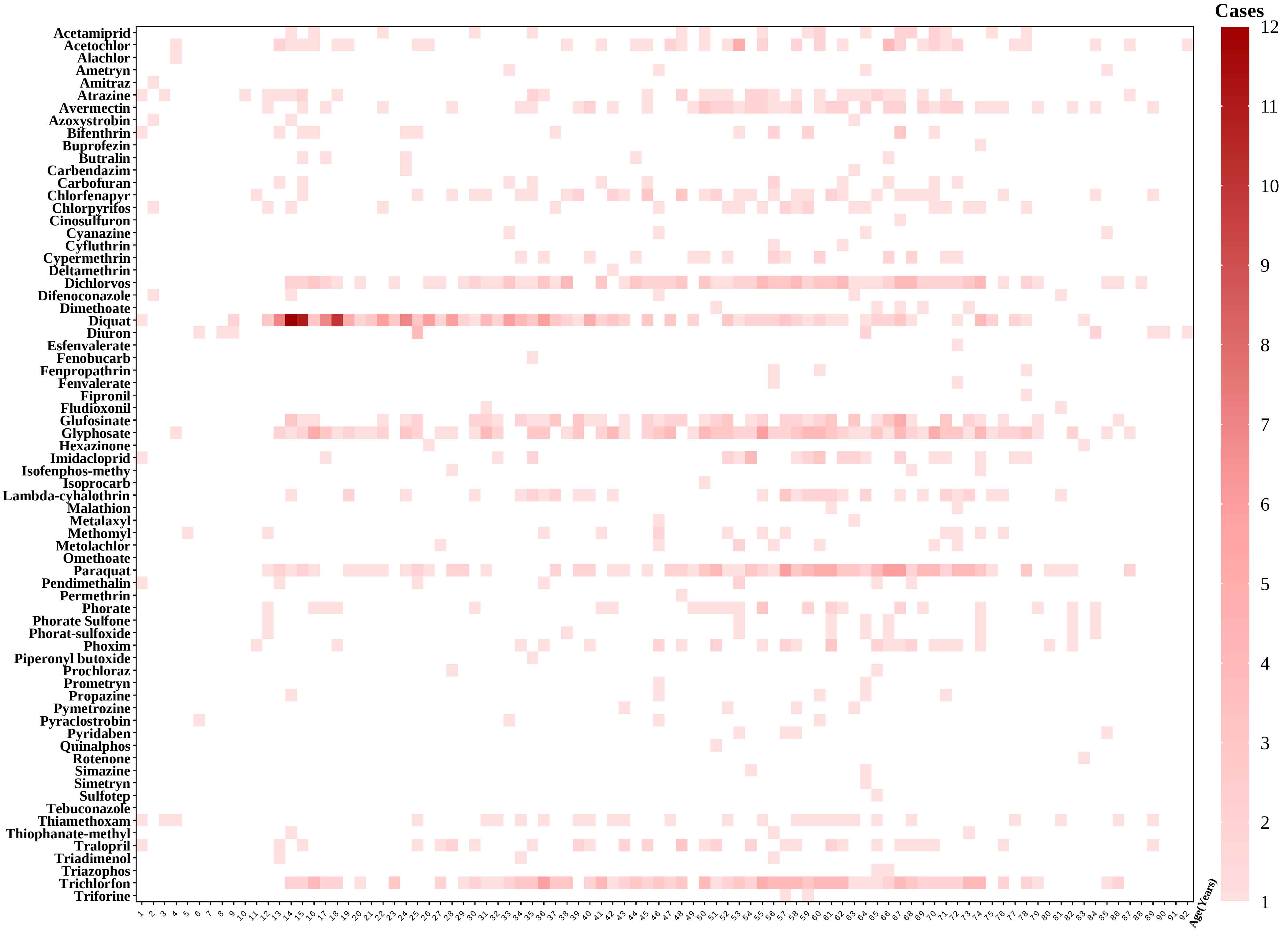

Cases
12
11
10
9
8
7
6
5
4
3
2
1
Acetamiprid
Acetochlor
Alachlor
Ametryn
Amitraz
Atrazine
Avermectin
Azoxystrobin
Bifenthrin
Buprofezin
Butralin
Carbendazim
Carbofuran
Chlorfenapyr
Chlorpyrifos
Cinosulfuron
Cyanazine
Cyfluthrin
Cypermethrin
Deltamethrin
Dichlorvos
Difenoconazole
Dimethoate
Diquat
Diuron
Esfenvalerate
Fenobucarb
Fenpropathrin
Fenvalerate
Fipronil
Fludioxonil
Glufosinate
Glyphosate
Hexazinone
Imidacloprid
Isofenphos-methy
Isoprocarb
Lambda-cyhalothrin
Malathion
Metalaxyl
Methomyl
Metolachlor
Omethoate
Paraquat
Pendimethalin
Permethrin
Phorate
Phorate Sulfone
Phorat-sulfoxide
Phoxim
Piperonyl butoxide
Prochloraz
Prometryn
Propazine
Pymetrozine
Pyraclostrobin
Pyridaben
Quinalphos
Rotenone
Simazine
Simetryn
Sulfotep
Tebuconazole
Thiamethoxam
Thiophanate-methyl
Tralopril
Triadimenol
Triazophos
Trichlorfon
Triforine
1 2 3 4 5 6 7 8 9 10 11 12 13 14 15 16 17 18 19 20 21 22 23 24 25 26 27 28 29 30 31 32 33 34 35 36 37 38 39 40 41 42 43 44 45 46 47 48 49 50 51 52 53 54 55 56 57 58 59 60 61 62 63 64 65 66 67 68 69 70 71 72 73 74 75 76 77 78 79 80 81 82 83 84 85 86 87 88 89 90 91 92
Age(Years)
